# Stress signaler p38 mitogen-activated kinase activation: a cause for concern?

**DOI:** 10.1042/CS20220491

**Published:** 2022-11-14

**Authors:** Enkhtuya Radnaa, Lauren Richardson, Brett Goldman, Jared K. Burks, Tuvshintugs Baljinnyam, Natasha Vora, Hui-juan Zhang, Elizabeth A. Bonney, Arum Han, Ramkumar Menon

**Affiliations:** 1 Division of Basic and Translational Research, Department of Obstetrics and Gynecology, The University of Texas Medical Branch at Galveston, Galveston, Texas, U.S.A.; 2 Flow Cytometry and Cellular Imaging Core Facility, Department of Leukemia, M.D. Anderson Cancer Center, Texas, U.S.A. 77030; 3 Department of Pharmacology and Toxicology, The University of Texas Medical Branch at Galveston, Galveston, Texas, U.S.A. 77555; 4 Department of Pathology, The International Peace Maternity and Child Health Hospital, University School of Medicine, Shanghai, China. 200030; 5 Department of Obstetrics and Gynecology, The University of Vermont, Burlington, VT, U.S.A. 05405ghout all figures, the following notations were; 6 Department of Electrical and Computer Engineering, Department of Biomedical Engineering, Texas A&M University, College Station, Texas, U.S.A. 77843

**Keywords:** EMT, pregnancy, Senescence

## Abstract

Oxidative stress (OS) induced activation of p38 mitogen-activated kinase (MAPK) and cell fate from p38 signaling was tested using the human fetal membrane’s amnion epithelial cells (AEC). We created p38 KO AEC using the CRISPR/Cas9 approach and tested cell fate in response to OS on an AEC-free fetal membrane extracellular matrix (ECM). Screening using image CyTOF indicated OS causing epithelial–mesenchymal transition (EMT). Further testing revealed p38 deficiency prevented AEC senescence, EMT, cell migration, and inflammation. To functionally validate *in vitro* findings, fetal membrane-specific conditional KO (cKO) mice were developed by injecting Cre-recombinase encoded exosomes intra-amniotically into *p38α^loxP/loxP^* mice. Amnion membranes from p38 cKO mice had reduced senescence, EMT, and increased anti-inflammatory IL-10 compared with WT animals. Our study suggested that overwhelming activation of p38 in response to OS inducing risk exposures can have an adverse impact on cells, cause cell invasion, inflammation, and ECM degradation detrimental to tissue homeostasis.

## Introduction

Reactive oxygen species (ROS) generated in response to both endogenous and exogenous stimuli initiate various stress signaling responses that can determine cell fate which includes proliferation, transition, differentiation, senescence, and cell death [1,2]. Physiologic activation of p38 during the cell cycle, along with their network interaction with cyclin-dependent kinases can control DNA replication, and regulate cell proliferation, cellular transitions to help with growth and development [3]. Cellular organelle, DNA, and protein damage arising from exposure to various stress stimuli can cause p38 excess activation and in turn, lead to various pathologies [4]. This is evident in cell type-dependent growth-promoting or growth-suppressing activities as seen in certain tumorigenesis or pathologic aging, respectively. Though complex, balanced regulation of p38 activation and function is essential to maintain proper cell function [3,5,6].

Pregnancy provides one of the best states to study the mechanisms of p38 function and its implications. The distinct phases of cell fates are well-displayed during human pregnancy where the rapid growth of the fetus and fetal tissues occurs until approximately 9 months and a gradual decline in cellular activities due to senescence ensures fetal delivery at term [7–14]. p38 is expressed during the development of the fetal membrane (amniochorionic membranes) that lines the intrauterine cavity and which is constantly bathed in the amniotic fluid [10,11,13,15]. Fetal membranes’ structural and mechanical stability and antimicrobial properties are essential for fetal growth and pregnancy maintenance [13,16]. The pregnant uterine cavity experiences distinct oxidative environments at different trimesters where locally generated and tightly regulated ROS helps tissue growth and remodeling that is enabled by p38 [10,17]. Studies have shown that activated form of p38 at various gestational periods, primarily associated with cell cycle progression but also with progressive cellular senescence to facilitate biologic aging of fetal membranes. p38 functions in the intrauterine tissues are regulated by transforming growth factor (TGF)-b mediated activation, which is a constituent of the amniotic fluid [10,18–21]. TGF-b, by a noncanonical pathway, autophosphorylates p38 amnion epithelial cells (AEC) of the fetal membrane thus bypassing the traditional activation mechanisms that involve a cascade of MAP kinases activation. Cellular remodeling during pregnancy involves EMT of AEC, a mechanism that can generate localized inflammation and matrix regeneration to remodel the fetal membranes [19]. Regulated activation of p38 is also associated with these activities.

Structurally, the AEC layer is attached to the collagen-rich extracellular matrix (ECM) region via Type IV collagen-rich basement membranes [16]. Transitioned AEC comprise the major population of amnion mesenchymal cells (AMC) that complete the ECM scaffold. ECM-mediated mechanical forces play a major role in the ECM structural integrity, as well as control p38-mediated cell migration. Cellular transition, migration, and matrix invasion are like that seen in a tumor environment where p38 may play a prominent role [4,22]. However, the interactive role between the cells, the matrix, and p38 in this process is not fully characterized under normal conditions or in the presence of OS. Knowledge of p38’s function in AEC is important for understanding the pathophysiology of pregnancy complications such as spontaneous preterm birth and preterm premature rupture of the fetal membranes [9,10,13,23]. Both these conditions are associated with ROS-induced excessive p38 activation increase in cell damage, senescence, EMT, and inflammation [12,23–25].

Although descriptive analyses show p38’s engagement in pregnancy pathologies, the functional impact of p38 in cellular senescence, transition, cellular migration into the ECM layer, and generation of inflammation are not completely established [8,10,19,26]. To determine the critical role played by p38 in these processes, we created p38 deficient AEC using CRISPR/Cas9 technology and determined their properties under normal and OS environments. We first used a cell-free ECM (nude membrane lacking AEC layer) derived from normal term delivered human fetal membranes. Further, a mouse model of pregnancy where cKO of p38 in AEC was created to physiologically validate this model above by injecting Cre recombinase encoded extracellular vesicles (exosomes) intra-amniotically into *p38α^loxP/loxP^* mice. This model was created because generalized p38α knockout (null) results in embryonic lethality due to defective placental morphogenesis [27]. By disrupting the function of p38, these studies established a critical role played by stress signaler p38 in AEC senescence, EMT of AECs that does not undergo senescence and migration. EMT accumulates mesenchymal cells and produces an inflammatory environment that can further destabilize the membrane matrix [19,28,29].

## Methods

### IRB approval

Placental specimens were collected from John Sealy Hospital at the University of Texas Medical Branch at Galveston (UTMB), Texas, U.S.A. under a protocol to use discarded and de-identified placenta after normal term cesarean deliveries (UTMB 11-251). Research has been carried out in accordance with the World Medical Association Declaration of Helsinki. No subjects were recruited or consented to the present study. The present study falls under the category of human subject specimen defined by the National Institutes of Health. The present study used only discarded human specimens.

### Amnion membrane isolation

All tissue samples were collected from women undergoing elective repeat cesarean delivery (between 37 and 41 weeks gestation), before spontaneous labor and membrane rupture. Women were excluded if they had a history of preterm delivery, premature rupture of membranes, pre-eclampsia, placental abruption, fetal growth restriction, gestational diabetes, Group B streptococcus carrier status, history of treatment for a urinary tract infection during pregnancy, sexually transmitted infections during pregnancy, chronic infections such as HIV and hepatitis, or a history of cigarette smoking or drug and alcohol abuse. A periumbilical midzone portion of the fetal membrane (4 cm × 4 cm) was used from the human whole fetal membrane. The amnion layer was then separated from the chorion layer by peeling off manually as described previously [30]. The separated amnion membrane was then rinsed with prewarmed saline (0.9% sodium Chloride, Baxter, Deerfield, IL, U.S.A.).

### Amnion membrane *ex vivo* culture

Isolated amnion membrane was cultured in complete keratinocyte serum-free media (KSFM - Gibco #17005042) supplemented with human recombinant epidermal growth factor (rEGF, #10450-013, 2.5 µg), bovine pituitary extract (BPE, #13028-014, 25 mg), and primocin (Invitrogen, Carlsbad, CA, U.S.A., Cat #cat-pm-1, 50 mg/mL) in a 37°C, 5% CO_2_ incubator. In some experiments, a fluorescent probe Col-F (10 µM, ImmunoChemistry Technologies, Bloomington, MN, U.S.A., Cat. No: 6346) was used to label collagen and/or elastin in the ECM of amnion membrane overnight in a 37°C, 5% CO_2_ incubator before conducting experiments.

### Reconstructing amnion membrane *in vitro* using a transwell system

#### Immortalized AEC cell culture

Human primary AEC were isolated from the fetal membrane and immortalized as described previously [31]. Briefly, primary AEC were infected with human papillomavirus (HPV) type 16 E6E7 (HPV16E6E7) retrovirus (ATCC® CRL-2203™), and un-infected primary AEC were eliminated with 50 µg/ml of G418 (Corning®) for 5 days. Immortalized AEC were cultured in complete KSFM in a 37°C, 5% CO_2_ incubator. AEC were then transduced with pCT-CD9-RFP lentivirus (SBI, CA, U.S.A.) as previously described [32] to generate red fluorescent protein (RFP) stably expressing cell line (AEC-RFP) in the purpose of tracking the cells migration in the reconstructed amnion membrane.

#### p38 knockout (KO) in AEC-RFP cells using Clustered regularly interspaced short palindromic repeats (CRISPR)/CRISPR-associated protein 9 (Cas9) gene editing

To study the effect of the p38 in the EMT of the AEC in the amnion membrane, *MAPK14 (*p38α) was knocked out by the CRISPR/Cas9 gene-editing system in AEC-RFP. Multi-guide sgRNAs for *MAPK14* and Cas9 2NLS nuclease (*Streptococcus pyogenes* Cas9 protein with two nuclear localization signals) were purchased (Synthego Corp., Redwood City, CA, U.S.A.). Human *TRAC* multi-guide sgRNA (Multi-guide Transfection Optimization Kit, Synthego Corp.) was used as a positive control. The transfection of ribonucleoprotein (RNP) complexes including multi-guide sgRNAs (Synthego Corp.) and Cas9 nuclease (Synthego Corp.) was accomplished according to the manufacturer’s instructions using Lipofectamine™ CRISPRMAX™ Transfection Reagent (Invitrogen, Cat. No: CMAX00015). Briefly, the RNP mixture was prepared by dissolving 1.3 μl of multi-guide RNAs (3.9 pmol), 1 µl of Cas9 (3 pmol), and 1 µl of Lipofectamine™ Cas9 Plus Reagent into 25 µl of Opti-MEM™ (Gibco™, Cat. No: 31985-070) for 5 min at room temperature (RT). In some experiments, two times concentrated RNP mixture was prepared to find the optimal experimental conditions. The transfection solution mixture was then prepared by dissolving 1.5 µl of Lipofectamine™ CRISPRMAX™ Transfection Reagent into 25 μl Opti-MEM™ for 5 min at RT. Then, the RNP and transfection solution mixtures were incubated together for an additional 10 min at RT and added to 80,000 cells containing Eppendorf tubes. The reverse transfected cells were then seeded into 24-well plates and cultured in a 37°C, 5% CO_2_ incubator until to reach 85% of confluency. The expanded cells were analyzed for p38 KO efficiency with Western blot for p38 expression for every three passages and used for presented experiments. CRISPR/Cas9 gene-edited KO cell lines are the pooled cells. The positive control *TRAC* KO efficiency was analyzed with polymerase chain reaction (PCR) using the kit-supplied primer sets (forward: 5′-TCAGGTTTCCTTGAGTGGCAGG-3′; reverse: 5′-TAAGGCCGAGACCACCAATCAG-3′) according to the manufacturer’s instruction. Genomic DNA was isolated using QIAamp DNA Mini Kit (QIAGEN, Venlo, Netherlands, Cat. No: 51304) with the manufacturer’s instructions. *GAPDH* (forward: 5′-ACCACAGTCCATGCCATCAC-3′ and reverse: 5′-TCCACCACCCTGTTGCTGTA-3′) was run for the internal control.

#### Nude membrane preparation

To study AEC’s transition (EMT) due to OS, the amnion membrane was reconstructed *in vitro*. First, AEC were removed from the amnion layer with 5 M of urea (Mallinckrodt, Staines, U.K.)/1× phosphate-buffered saline (PBS, Corning™, Corning, NY, U.S.A., Cat. No: 21-031-CVR) treatment on ice for 5 min. AEC were scraped off with the cell scraper (Corning®, Cat. No:3010) under a dissection microscope. The nude membrane without AECs may still contains a few stromal cells (normally 10% of AECs) in the ECM [19]. was then gently rinsed with cold 1× PBS. For further experiments, nude membranes were attached to the transwell inserts after removing the polyethylene terephthalate (PET) membrane (Falcon®, Corning®, Cat. No: 353095) with sharp forceps. The nude membranes were attached to the inserts of the trans-well plates using UV-sterilized mini plastic bands with the basement membrane facing up and the rest of the ECM facing down.

#### Seeding wild type (WT) and p38 KO AEC-RFP on to the nude membrane

Approximately, 120,000 WT or p38 KO AEC-RFP cells were seeded onto a nude membrane to reconstruct the amnion membrane *in vitro*. The seeded cells were allowed to settle down on the membrane. All the experiments including treatment with OS inducing agents were performed after a resting period of 1 h.

### OS treatment

To induce OS, the tissues were treated with cigarette smoke extract (CSE). A water-soluble portion of CSE was prepared as previously described [30] and used at 1:25 dilution with complete media for all treatments. CSE as a lab reagent for inducing OS has provided consistent and reproducible data as well as OS-induced changes in AEC, which mimicked the same histologic changes observed in fetal membranes from preterm birth and premature rupture of the membranes.

### Western blot analysis

Immunoblotting was done as previously described with some modifications [32]. Briefly, samples were lysed with a 1× radioimmunoprecipitation assay (RIPA) lysis buffer (0.5 M Tris, pH 8.0; 1.50 M NaCl: 10% [v/v] Triton X-100; 10 mM EDTA, pH 8.0; and 10% [w/v] sodium dodecyl sulfate-SDS) supplemented with protease (Millipore, Sigma), phosphatase inhibitor cocktails (Thermo Scientific), and phenylmethylsulfonyl fluoride (PMSF, Honeywell Fluka, Charlotte, U.S.A.) [32]. A Pierce BCA Protein Assay Kit (Thermo Scientific) was utilized to determine the protein concentration of samples. About 20 μg total proteins were loaded into each well of 4–15% gradient polyacrylamide gels (Bio-Rad) and subjected to electrophoresis. Subsequently, the proteins were transferred to polyvinylidene fluoride (PVDF) membranes (Bio-Rad) by semi-dry electrophoretic transfer (Bio-Rad), and the membranes were blocked in 5% nonfat dry milk/TBST (Tris-buffered saline, 0.1% Tween20) for 2 h at RT. The membranes were then incubated with primary antibodies of p-p38 (Cell Signaling, Danvers, MA, U.S.A., Cat. No: 4511S, Lot No: 10, DF: 1:300), p38 (Cell Signaling, Cat. No: 9212S, Lot No: 26, DF: 1:1000) and β-actin (Sigma, Cat. No: A5441-2ml, Lot No: 079M4799V, DF: 1:15,000) diluted in 5% nonfat dry milk/TBST overnight at 4 °C, with secondary antibodies (Southern Biotech, Birmingham, AL, U.S.A., Cat. No.: 1030-05, Lot: K3515-T566, DF: 1:15,000) for 1 h at RT. Protein bands were visualized using an enhanced chemiluminescent solution (Bio-Rad) with ChemiDoc™ Imaging System (Bio-Rad). For immunoblot quantitative analysis, Image Lab™ software was used (Bio-Rad).

### Imaging mass cytometry analysis

#### Panel designing

Imaging mass cytometry antibody panel was designed to determine various stress-activated kinases, cytoskeletal, senescence, and EMT associated markers (Supplementary Table S1).

#### Antibody labeling

The following antibodies were labeled using the Maxpar® X8 multimetal labeling kit (Fluidigm Corporation, South San Francisco, CA, U.S.A., Cat. No: 201300, Lot No: MKSO-008656) according to the manufacturer’s instructions: P-p38 (Abcam, Cambridge, MA, U.S.A., Cat. No: ab236527), P-JNK (Invitrogen, Cat. No: 700031), integrin-β1 (Novus Biologicals, Littleton, CO, U.S.A., Cat. No: NBP2-22191), SNAI1 (Invitrogen, Cat. No: 14-9859-82), twist2 (Novus Biologicals, Cat. No: H00117581-M01), Zeb1 (Novus Biologicals, Cat. No: NBP2-81015), CD9 (Novus Biologicals, Cat. No: NB500-327), RFP (Novus Biologicals, Cat. No: NBP1-69962), and MMP9 (Novus Biologicals, Cat. No: NBP2-80855). Conjugated antibody concentration was assessed with a microplate spectrophotometer (280 nm, Synergy H4 Hybrid Reader, BioTek Instruments, Winooski, VT, U.S.A.) and adjusted to 500 μg/ml in antibody stabilizer PBS supplemented with 0.05% sodium azide (CANDOR Bioscience GmbH, Wangen im Allgäu, Germany, Cat. No: 131050). The remaining purchased antibodies were pre-conjugated commercially at Fluidigm: pan-cytokeratin (Fluidigm, Cat. No: 3148022D), CD98 (Fluidigm, Cat. No: 3173018D), and Mucin 1/CD227 (Fluidigm, Cat. No: 3150032D), and at Flow Cytometry and Cellular Imaging Core Facility, M. D. Anderson’s Cancer Center: E-cadherin, EpCAM, α-SMA, Vimentin, N-cadherin, β-catenin, and p-ERK (Supplementary Table S1) from the reagent repository. All metal-tagged antibodies were stored at 4°C. Antibody concentration and specificity were evaluated by visual inspection of the imaging mass cytometry images of OS-treated intact amnion membrane tissues using an MCD Viewer Software (Fluidigm). The resulting staining patterns were compared to the standard results presented by the various antibody vendors, various publications (BenchSci.com), and the Human Protein Atlas (https://www.proteinatlas.org/).

#### Sample preparation and imaging mass cytometry staining

Reconstructed amnion membranes were formalin-fixed and paraffin-embedded (FFPE). Tissue microarrays (TMAs) were prepared at the Anatomic Pathology Laboratory, Surgical Pathology Division of UTMB. Briefly, 0.6–0.8 mm long reconstructed amnion membranes were arranged 0.4 mm apart vertically in one cassette manually. One set of TMA cassettes contained four tissue pieces. About 5 µm FFPE tissue sections were prepared for imaging mass cytometry. Imaging mass cytometry staining was done as previously described with modifications [33]. Briefly, TMAs were deparaffinized in xylene and rehydrated in a graded series of alcohol. Heat-induced epitope retrieval was conducted in Tris (pH 9.0) (Agilent, Santa Clara, CA, U.S.A., Cat. No: S236884-2) using an Antigen Retriever 2100 according to manufacturer’s instructions (Aptum Biologics, Southampton, U.K.). Tissues were then blocked with 3% BSA/1× TBST for 1 h at RT and incubated with antibody cocktails diluted in 3% BSA/1× TBST overnight at 4°C. Antibody concentrations for the staining were described in Supplementary Table S1. The next day, tissues were washed three times with 1× TBST for 10 min each and then incubated with 125 nM of Cell-ID™ Intercalator-Ir (Fluidigm, Cat. No: 201192A, Lot No: 2104787-23) /1× PBS for 1 h at RT for nuclear staining. Tissues were rinsed with milli-Q H_2_O for 10 min at RT. Lastly, tissues were air-dried for 30 min and stored at RT until ablation.

#### Imaging mass cytometry ablation

Images were acquired using a Hyperion mass cytometry system (Fluidigm). Laser-ablation of the stained TMAs was conducted at the Flow Cytometry and Cellular Imaging Core Facility, M.D. Anderson Cancer Center (North Campus). The Hyperion was autotuned using a 3-element tuning slide (Fluidigm) according to the tuning protocol provided by the imaging system user guide (Fluidigm). Regions of interest were selected based on a provided tissue map that selected three regions of interest that equaled 1000 × 1000 µm per tissue section. Three biological replicates were images for each category of the reconstructed membrane (control or OS treated) containing WT or p38 KO AEC cells. Ablation was conducted at 200 Hz and raw images were collected.

#### Imaging mass cytometry data analysis

Raw data were exported as MCD files and visualized using the Fluidigm MCD™ Viewer (https://www.fluidigm.com/products-services/software). Individual image files for each stain and region of interest were exported from the MCD™ Viewer as a 16-bit multilayer TIFF file. CellProfiler (https://cellprofiler.org/) was used to conduct cell segmentation and generate a cell mask. A pipeline was built focusing on the nuclear stain (Cell-ID™ Intercalator-Ir, Fluidigm, Cat. No: 201192A, dilution factor 1:500) as the primary object (5–20 pixels) and 3 pixels outside the primary object the cytoplasm mask was created. All files were run through this pipeline and cell masks were generated for each tissue and region of interest. The TIFF files and cell masks were loaded into HistoCat (https://bodenmillergroup.github.io/histoCAT/) for phenotype analysis. Each tissue category (three regions of interest for three replicates) was analyzed together in the same pipeline. All marker expression data per category was visualized as a t-SNE plot. Phenograph analysis was conducted using standard HistoCat settings to generate unique cell clusters from each category. Cluster identification was achieved using the generated heatmaps for antibody intensity. Clusters of interest were determined if they met the following criteria: (1) contained nuclear antibody expression, (2) targets that were below the threshold for determinization of clear identity, or (3) expressed p38 in a known KO line. Clusters that met all the above criteria were analyzed for pathways of EMT which are indicative of phenotype and migratory potential. Cut-off value 0.6 was set determines the positive expression of markers from the heat map to determine the cell fate. Our hypothesis was driven from this preliminary analysis of cell fate that is displayed in the summary table. Further characterization of cells and their changes were conducted based on these expected changes.

#### Immunohistochemistry (IHC) analysis on frozen sections

After treatments, the tissues were transferred to Tissue-Tek optimal cutting temperature (O.C.T, Sakura Finetek, Tokyo, Japan) compound to snap freeze for immunohistology. IHC was done as previously described [32,34]. Briefly, 10 µm tissue sections were air-dried on the slide glasses (Matsunami Glass, Osaka, Japan) for 40 min at RT, then washed with tris-buffered saline with 0.1% Tween20 (1× TBST) for 10 min. Tissues were blocked with 3% bovine serum albumin (BSA Gemini, Bio-Products, West Sacramento, CA, U.S.A.)/1× TBST for 1 h followed by overnight incubation with vimentin diluted at 1:300 (Abcam, Cat. No.: ab92547, Lot No.: GR3186827-13) and cytokeratin-18 diluted at 1:800 (Abcam, Cat. No: ab668, Lot No: GR3196069-6) in 3% BSA/1× TBST at 4°C. The next day, tissues were washed 3 times with 1× TBST for 10 min each, then treated with secondary antibodies diluted at 1:1000 (Alexa Fluor® 488, Abcam, Cat. No.: ab150073, Lot: GR269274-4 and Alexa Fluor™ 594, Invitrogen, Cat. No.: A11005, Lot: 2179228, respectively) in 3% BSA/1× TBST for 3 h at RT. Then, tissues were stained with NucBlue™ Fixed Cell ReadyProbes™ (DAPI, Invitrogen, Cat. No: R37606, Lot No: 2216969) for 5 min, and washed 3 times with 1× TBST for 10 min each. The images were taken with a Keyence microscope (Keyence Corp., Osaka, Japan).

#### Z-stack time-lapse analysis of the AEC-RFP migration

RFP-labeled AEC migration through the ECM of the amnion membrane was monitored via Z-stack time-lapse imaging using a KIW chamber (37°C, 5% CO_2_) for Keyence microscope (Keyence Corp.). Reconstructed amnion membrane on the trans-well system was placed in the environmental chamber of the microscope (Keyence Corp.) and AEC-RFP was imaged through Z-Stack time-lapsing. Time-lapse images were analyzed using the 3D Measure function, BZ-X800 Analyzer software (Keyence Corp.).

#### Transforming growth factor-β (TGF-β) induced EMT in AEC-RFP

TGF-β was used to induce EMT in WT and p38 KO AEC-RFP. Approximately 70,000 cells were seeded in a 24-well plate overnight to ensure the attachment of the cells. The cells were then treated with 5, 15, and 20 ng/ml of TGF-β (R&D Systems, Inc., Cat. No: 240-B-002), and cultured in a 37°C, 5% CO_2_ incubator. The media were replaced every other day, and cells were imaged 7 and 10 days after the initial treatment.

#### CSI analysis

CSI, a quantitative measure of cell morphology acquired from images, of WT and p38 KO AEC-RFP cells upon the TGF-β treatment was carried out as previously described [19,35]. Briefly, imaged CSI was determined using ImageJ software (three images for each treatment). The shape index of 1 was evaluated as a circle, as 0 was evaluated as a straight line. The shape index was calculated using the formula of SI = 4π*Area/Perimeter^2^ [35] (https://imagej.nih.gov/ij/plugins/circularity.html).

#### Immunoassay for released cytokines

Multiplexed ELISA was performed to detect the human inflammatory cytokine levels in the reconstructed amnion membrane model in the transwell system. Culture media from the top part of the transwell and the bottom part of the transwells were collected separately from 24-transwell. ELISA was performed using a MILLIPLEX® human cytokine panel for interleukin (IL)-6, IL-8, IL-10 and Granulocyte-macrophage colony-stimulating factor (GM-CSF) (Millipore, Burlington, MA, U.S.A.) according to manufacturer’s instructions as described previously [36,37] with the MILLIPLEX™ Analyzer (Luminex® Corp., Austin, TX, U.S.A.). For mice samples, serum was collected by centrifuging at 2000 ***g*** for 10 min after incubating on ice for 30 min. Tissue samples were lysed with RIPA lysis buffer as described above. ELISA was performed using a MILLIPLEX® mouse cytokine panel for IL-6, IL-8, IL-10, and GM-CSF (Millipore).

### p38 cKO mice generation using cyclic-recombinase (Cre)-containing engineered exosomes

#### p38α^loxP/loxP^ mice

All animal procedures were approved by the Institutional Animal Care and Use Committee (IACUC) at the University of Texas Medical Branch, Galveston (Protocol number: 0411077E). All animal works were conducted at the University of Texas Medical Branch under the protocols approved by the University IACUC. Mice were housed in a temperature- and humidity-controlled facility with 12:12-h light/dark cycles. To mimic the human amnion membrane study *in vivo*, p38 cKO mice were generated using *p38α^loxP/loxP^* mice (Jackson Lab, Bar Harbor, ME U.S.A., Stock: *Mapk14tm1.2Lex/YiwaJ*, Stock No: 031129) combination with the engineered exosomes that contain Cre. *p38α^loxP/loxP^* mice were purchased, and Cre-exosomes was kindly gifted from *ILIAS Biologics*, Inc. (Daejeon, Republic of Korea) [38]. *p38α^loxP/loxP^* mice possess *loxP* sites flanking exon 1, including the start codon, of the mitogen-activated protein kinase 14 (*Mapk14*) gene in C57BL/6NJ background [39]. Mice were bred several generations and genotyped as recommended (Jackson Lab). Briefly, genomic DNA samples were prepared from 1 mm of snipped tails using the mouse tail DNA extraction reagent (101 Bio, Mountain View, CA, U.S.A., Cat. No: T605) according to the manufacturer’s instructions. PCR was conducted using the REDTaq® ReadyMix™ PCR Reaction Mix (Sigma Aldrich, St. Louis, MO, U.S.A., Cat. No: R2523) with the following condition: 25 µl of REDTaq® ReadyMix, 2 µl of 1 µM primer set (Forward: 5′-AGC CGC GTC CCT CTT CTC- 3′; Reverse: 5′-GGA GCC TCT CGC GGA ACT- 3′), 4 µl of genomic DNA, and nuclease-free H_2_O up to 50 µl in total. The PCR amplification was conducted using an iCycler thermal cycler (Bio-Rad Laboratories, Inc., Hercules, CA) with the following conditions: 2 min at 94°C denaturation followed by 30 cycles at 94°C for 20 s, 55°C for 30 s, and 72°C for 1 min. The PCR products (∼200 bp for mutant, 150 bp for WT) were then analyzed with 1.5% agarose gel electrophoresis. *p38α^loxP/loxP^* females (8–12 weeks old) were mated with males of the same genotype (*p38α^loxP/loxP^*) for homozygous embryos. Female mice were checked for the appearance of a vaginal plug, which indicated 0.5 days post coitus (DPC). The weight of the females was monitored, and a gain of 1.75 g by E10.5 confirmed pregnancy [38]. E13 pregnant *p38α^loxP/loxP^* mice were intra-amniotically injected with Cre-recombinase exosomes as previously described [32]. Briefly, embryos on the left side of the horn were injected with 25 µl of sterile PBS (endotoxin-free Dulbecco’s PBS (1×), w/o Ca++ and Mg++, MilliporeSigma, Cat. No: TMS-012-A) for the control and the right side of the horn were injected with 25 µl of Cre-exosomes (1.00E+10 per embryo) to conditionally knockout the p38 in the fetal membrane. Twenty-four hours after the injection, the tissue samples were collected for Western blot (snap freeze) and IHC (β-Gal staining on frozen sections). For a positive control for our Cre-exosome treatment, mT/mG mice (*Gt (ROSA)26Sortm4 (ACTB-tdTomato,-EGFP)Luo/J* in C57BL/6J background, Stock No. 007676) were used with the same condition. These mice were previously reported by our group [38]. For some experiments, E13 *p38α^loxP/loxP^* mice were intra-amniotically injected with Cre-exosomes (25 µl of 6.75E+08 exosomes per embryo) for both horns to conditionally knockout the p38 in the fetal membrane and sacrificed at E18 by CO_2_ asphyxiation. Naïve exosomes (25 µl of 6.75E+08 exosomes per embryo) were injected as controls. Maternal body weights were measured each day to monitor embryonic growth. At E18, collected tissues were homogenized using a bullet blender (Next Advance, Averill Park, NY) as previously described [40] for Western blot and multiplexed ELISA.

#### Engineered Cre-exosome preparation and characterization

Cre-exosomes were kindly gifted to us from *ILIAS Biologics* Inc. (Daejeon, Republic of Korea), and its production was previously described [38]. Briefly, HEK293T cells were transfected with Cre-mcherry-CRY2 and CIBN-EGFP-CD9 expression vectors. Stable cell lines were generated from single cells with red (mCherry) and green (GFP) fluorescence that were cultured until confluence. Cells were incubated with serum-free medium for 48 h under 488 nm blue-light illumination. The medium, as well as medium from HEK293T cells cultured under standard conditions (naive), was collected, and filtered through a syringe filter (0.2 μm, Sartorius, Goettingen, Germany) to remove cellular debris. Exosome isolation and purification were performed by tangential flow filtration and size exclusion chromatography. Exosome particles’ size distribution and concentrations were measured by nanoparticle tracking analysis (ZetaView, Particle Metrix, Germany) as described previously [37]. The surface and internal markers of the exosomes were analyzed using the ExoView® Tetraspanin Kit (NanoView, Boston, MA, U.S.A.) as previously described with minor modifications [32]. Briefly, 35 μl of exosomes (1 × 10^9^ /ml) were diluted in 1× Solution-B (NanoView™ Biosciences), applied on to the tetraspanin microarray chips, and incubated at RT overnight. The unbound exosomes were washed three times for 3 min at 500 rpm shaker in solution A. CD9 and CD63 antibodies/blocking solution (NanoView Biosciences) were incubated with the exosomes for 1 h at RT in the dark. The chips were then sequentially washed and imaged on the ExoView™ R100 instrument (NanoView™ Biosciences) using the nScan 2.9.3 acquisition software. Cre-exosomes contain mCherry-fused Cre, therefore, Cre-recombinase loading efficiency was detected via mCherry signals. The size distribution, surface tetraspanin, and the internal Cre-loading efficiency were calculated using NanoViewer 2.9.3. Cre-exosomes contain Cre protein, not mRNA.

### Senescence staining

Senescence staining was performed according to the manufacturer’s instructions using a Senescence Cells Histochemical Staining Kit (Sigma Aldrich, Cat. No: CS0030) and we reported previously [25]. Briefly, cells were fixed and stained with an X-gal staining mixture for 37°C for 3 h. Frozen tissue samples were prepared as described above. About 10 µm thick sections of mouse tissues were air-dried on the slide glasses (Matsunami Glass, Osaka, Japan) for 40 min, surrounded with a pap-pen, fixed with a fixation solution (Sigma Aldrich, Cat. No: CS0030) for 20 min, washed with 1×P BS for 10 min at RT. Tissues were then incubated with an X-gal staining mixture (Sigma Aldrich, Cat. No: CS0030) for 37°C for 24 h. Then tissues were washed with PBS for 10 min, stained with NucBlue™ Fixed Cell ReadyProbes™ (DAPI, Invitrogen, Cat. No: R37606, Lot No: 2216969) for 5 min, and washed three times with 1× PBS for 10 min each. Samples were imaged using a Keyence microscope (Keyence corp.) via bright field imaging.

### Statistical analysis

Statistical analysis was performed using the GraphPad Prism 8.0 software (GraphPad, San Diego, CA). Statistical parameters associated with each figure are reported in respective figure legends. All data are reported as mean ± SEM. Statistical significance in differences between experimental groups was assessed as follows: two-way ANOVA for immunoblot quantitative analysis, Unpaired *t*-test and two-way ANOVA for senescence analysis and cellular migration rate using 3D measure analysis, Multiple comparisons one-way ANOVA for CSI analysis, and two-way ANOVA for multiplexed ELISA. Throughout all figures, the following notations were used: *P<0.05, **P<0.01, and ***P<0.001. Significance was considered at *P*<0.05.

## Results

### Reconstructing the amnion membrane with p38 KO cell lines

**Figure 1:**
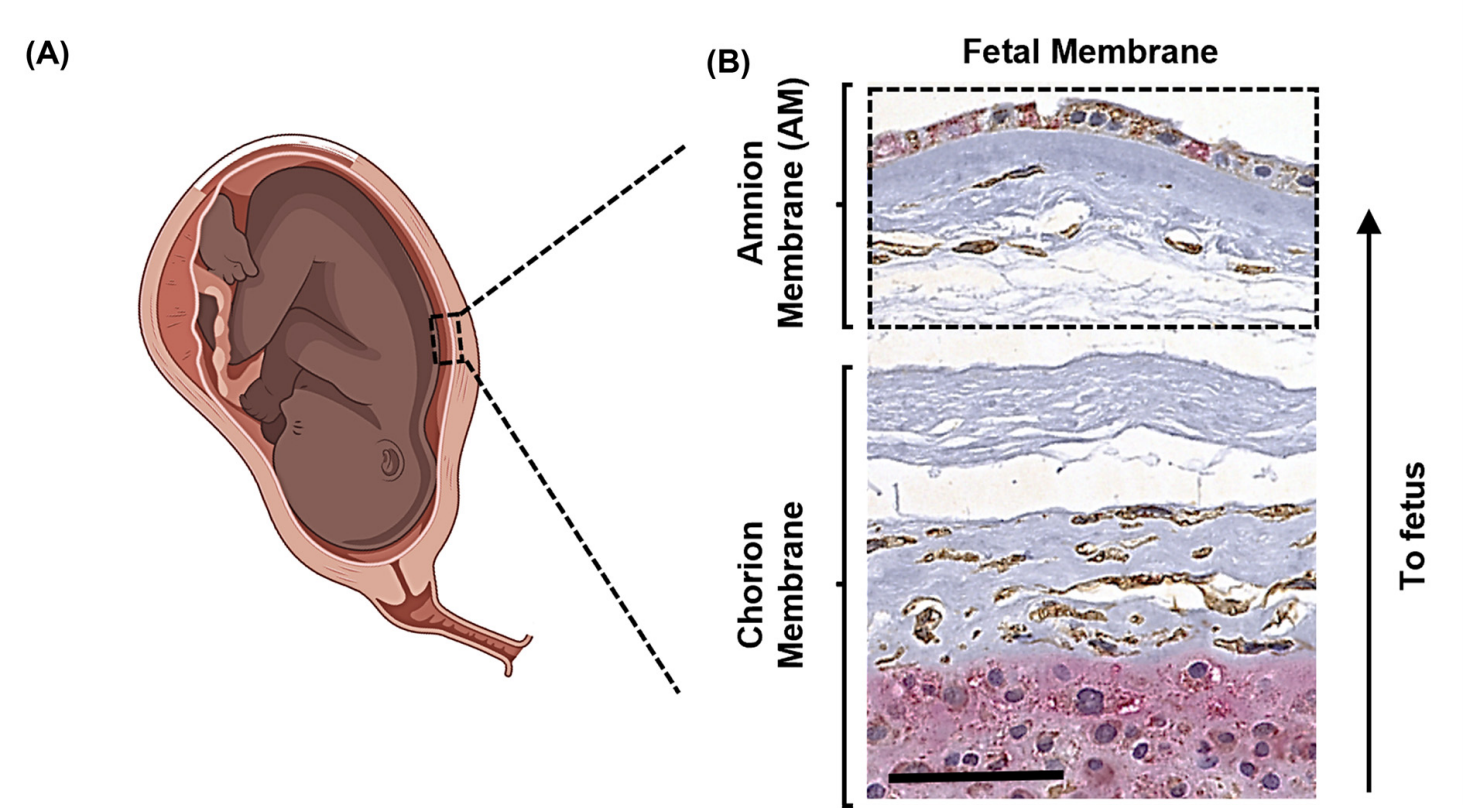
A model system establishment to study the p38 role in EMT and inflammation (**A**) A cartoon illustration of a dashed box displaying the fetal membrane location in the human embryo. (**B**) A Representative image showing human fetal membrane structure. An arrow is displaying the orientation of the organ in the embryo. Immunohistochemical staining displaying vimentin (brown) and cytokeratin-18 (pink) using 5 µm thick FFPE tissue sections from normal human fetal membrane. Hematoxylin (blue) is showing the nuclear staining. Amnion membrane was indicated as an AM in dashed box.

Activation of p38 induces multiple disease states in different organ systems [5]. To study the role of p38 activation and its contribution at the cellular level, we utilized the fetal membranes (Figure 1A,B) as a model tissue. p38 activation is associated with cellular senescence, EMT, migration, ECM degradation, and inflammation contributing to preterm birth. The amnion component of the fetal membranes (Figure 1B) is ideal to study p38’s role in the induction of either a cancer phenotype (gestational period) or aging pathologies (term pregnancy before delivery). Amnion membrane contains a layer of AEC connected to an ECM containing AMC (Figure 1B) through a basement membrane. This layer is constantly going through cellular and collagen remodeling to maintain homeostasis [19]. The overall goal of the present study is to determine the mechanistic role of p38 in causing the changes in AEC to confirm its association with the senescence of AEC and EMT.

**Figure 2:**
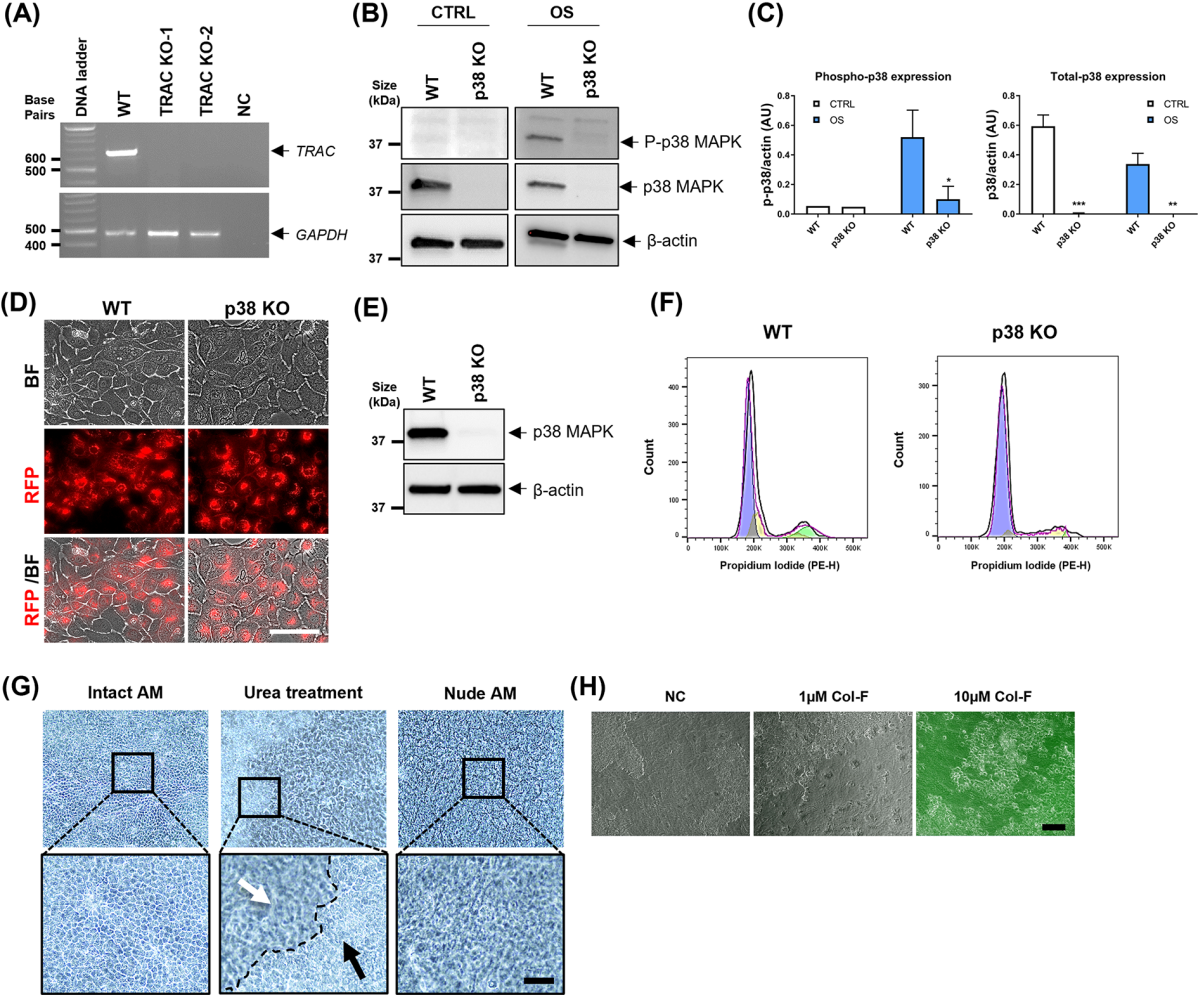
Reconstructing amnion membrane using p38 KO AEC-RFP and nude membrane (**A**) CRISPR/Cas9 transfection condition optimization in AEC-RFP cells using *TRAC* gene deletion. *TRAC* gene was successfully knocked out two different transfection conditions (x1 and x2, see Methods section) using CRISPR/Cas9 system. PCR was conducted 7 days after the transfection. Agarose gel electrophoresis images showing PCR product for *TRAC* gene (667 bp) which was detected in WT samples, but not in KO samples. *GAPDH* (460 bp) was run for internal control. NC, negative control. (**B**) Western blot analysis for validating *MAPK14* (p38) CRISPR KO efficiency with 20 µg of total protein lysates from AEC-RFP. Cells were treated with OS for 3 h to activate p38. p38 KO cell line showing significantly diminished phospho- and total- p38 expression compared with WT cells. P-p38 and p38 protein bands are normalized to β-actin with semi-quantitative densitometry analysis (**C**) using an Image Lab™ software. β-Actin was detected as a loading control (*N*=5). The data are presented as the means ± SEM; *P=0.034, **P<0.01, ***P<0.001. Multiple comparison two-way analysis of variance (ANOVA); OS, oxidative stress. (**D**) Representative images showing the morphology of the WT and p38 KO AEC-RFP cells; scale bar: 100 µm, BF, bright field. (**E**) p38 complete KO confirmation at passage 9 (P9) by comparing WT and p38 KO AEC-RFP cell lines. Western blot analysis for total p38 expression using 20 µg of total protein lysates. β-Actin was detected as a loading control. (**F**) Cell cycle confirmation of the p38 KO AEC. Untreated control p38 KO cells show comparable cell cycle pattern to WT AEC (*N*=3). (**G**) Removing natural AEC sheet from the amnion layer with 5 M urea treatment. AEC layer of the amnion membrane from the top view. Intact AM: untreated normal AEC sheet. Urea treatment: AEC detachment in process, before (black arrow) and after (white arrow) removal of native AEC from the amnion membrane. Nude AM: acellular amnion membrane after complete removal of the AEC sheet (*N*=5); scale bar: 50 µm. (**H**) Nude amnion membrane labeled with a green-fluorescent dye, Col-F. Collagen and elastin in the ECM of the amnion membrane were stained well with 10 µM of Col-F solution overnight in a 37°C, 5% CO_2_ incubator, however not at lower concentration (1 µM). NC shows an unstained nude amnion membrane from the top view; scale bar: 50 µm, NC, negative control.

Supplementary Figure S1 shows the schematic illustration of experimental approaches to reconstruct the amnion membrane *in vitro* using WT and p38 KO AEC on a nude membrane using a trans-well system. First, we made an RFP-expressing stable AEC cell line by infecting the immortalized AEC [31] with CD9-fused RFP lentiviruses to track the movement of AEC through the nude membrane (Supplementary Figure S1, step 1). Next, p38α was knocked out with CRISPR/Cas9 using multi-guide RNA against *MAPK14* (Supplementary Figure S1, step 2, Supplementary Figure S2 and Figure 2A–E). To do this, the transfection condition of multi-guide sgRNA and Cas9 mixtures were optimized to establish our CRISPR/Cas9 system, and its KO efficiency using *TRAC* gene-based transfection optimization kit (Figure 2A). With AEC-RFP cells, two transfection conditions worked well to knockout the target *TRAC* gene. After optimizing the transfection conditions, p38 was knocked out in AEC-RFP under the same conditions. Figure 2B shows p38 CRISPR KO efficiency with Western blot analysis. OS-treatment for 3 h activated p38 in WT but not in p38 KO cells (Figure 2B,C). In addition, p38 total protein expression was not detected in p38 KO cells (Figure 2B,C). Further studies were carried out using these cells with confirmed p38 KO AEC-RFP. Before using these cells, their viability and morphology were also confirmed. As shown in Figure 2D, p38 KO AEC-RFP cells were viable and exhibited similar morphology comparable to WT cells under normal culture conditions. p38 CRISPR KO efficiency was checked through various passages of AEC and observed effective, complete KO at passage 9 (Figure 2E), a maximum passage used for our experiments. The cell cycle of the p38 KO cells showed comparable results (Figure 2F and Supplementary Figure S3) suggesting that p38 is not the sole regulator of AEC proliferation and that other signalers compensate for the loss.

To generate an amnion membrane devoid of p38, we removed the native AEC from the amnion membrane using a 5 M urea treatment (Supplementary Figure S1, step 3, and Figure 2G). Next, we checked the intactness of the matrix collagen before our experiments. For this, the nude amnion membranes were stained with green-fluorescent probe Col-F, which can bind to collagen and elastin in the ECM. This staining aided visualization of the amnion membrane which is naturally clear (Figure 2H).

### Cell fate of AEC after p38 KO

**Figure 3:**
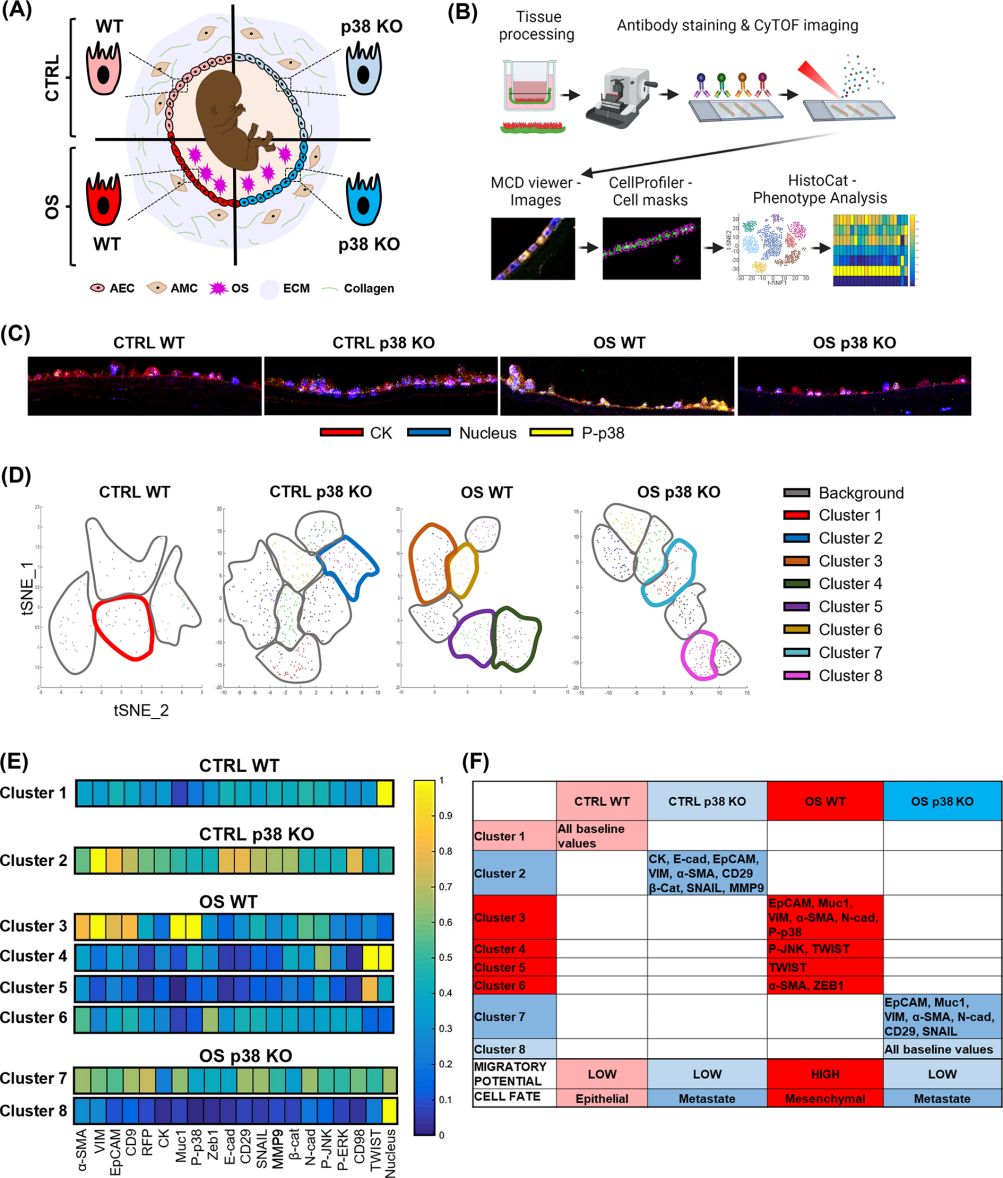
Analysis of p38 KO cellular fate and phenotypes using imaging mass cytometry (**A**) Cartoon representing the amnion compartment of the fetal membrane with WT and p38 KO AEC in CTRL *vs*. OS environment to determine their phenotype and fate using imaging mass cytometry technology. (**B**) Graphic schematic highlighting the imaging mass cytometry work flow: the reconstructed membranes were cultured within a transwell and then removed and sectioned onto a histology slide. These slides were stained with metal conjugated antibodies and images were captured through ablation. Images were analyized with MCD viewer, CellProfiler, and HistoCat to generate phenotype clusters. (**C**) MCD viewer processed imaging mass cytometry images showing P-p38 (yellow) expression in cytokeratin (CK; red) epithelial cells. Nuclear stain in blue. (**D**) t-SNE plots were generated for each category of reconstructed membranes and clusters were identidied through HistoCat phenotyping. (**E**) Clusters were analyized for cytoskeletal markers, transcription factors, and activated kinases by antibody intensity represented by a heatmap. (**F**) EMT markers and kinesis which expressed at or above 0.6 (threshhold value) on the heatmap are listed in the table.

The next set of experiments was conducted to decide the end phenotype measurements required in our experiments to determine the functional contribution of p38 in AECs. A comprehensive screening of reconstructed fetal membranes was conducted using imaging mass cytometry to precisely select additional tests. Although p38 KO did not impact AEC proliferation as demonstrated by multiple passages of our p38 KO cells, we were interested in measuring specific changes in cell signaling and true cell fate of AEC under normal (CTRL) and OS environment (Figure 3A). For this, reconstructed either WT or p38 KO AEC-RFP-containing amnion membranes were cultured under normal or OS environment for 24 h (Supplementary Figure S1, step 4). The membranes were then embedded in paraffin, sectioned on to glass slides, and were stained with a panel of 19 metal labeled antibodies (Figure 3B). To determine the cellular phenotypic status (‘epithelial state’ or ‘mesenchymal state’) and activation status of the AEC within the reconstructed membrane, imaging mass cytometry was performed to assess changes to cytoskeletal proteins, activation of stress kinases, and transcription factors (Supplementary Table S1). Imaging mass cytometry showed p38 activation in WT AEC with the reconstructed membranes after OS treatment compared to CTRL WT cell-containing membranes (Figure 3C). p38 activation was inhibited in p38 KO cell-containing membrane models (Figure 3C). To determine the global cellular profile, t-SNE plots were generated to identify neighboring cell populations (Figure 3D). Phenotype analysis of cells separated them into individual cell clusters 1-8. Each cluster of cells was unique based on how they were reconstructed on the nude membrane as well as their response to OS treatment (Figure 3D). Clusters identified by HistoCat that did not meet our inclusion criteria described in the Methods section were considered background. Cluster 1 from WT cell-containing membranes exhibited baseline expression of all markers suggesting that the WT AEC in the reconstructed membrane is in homeostasis under normal conditions (Figure 3E,F). Cells from p38 KO cell-containing membranes under normal conditions (i.e., cluster 2) showed an ‘intermediate state’ phenotype (‘metastate’) [41–43] expressing both epithelial (i.e., CK, EpCAM, E-Cad) and mesenchymal (i.e., vimentin) markers, which could be cellular function balancing act induced by p38 deficiency; however, they do not fully transition to mesenchymal phenotype (Figure 3E,F). Interestingly, transcription factor SNAI1 was detected in p38 KO cells in the reconstructed membrane in both normal and OS environment (Figure 3E,F). Recently, Kalluri’s group reported that SNAI1 complete KO had no effect on EMT in cancer progression and metastasis using mouse model of pancreatic ductal adenocarcinoma [42,43]. Therefore, it is arguable that SNAI1 in p38 KO cells could be compensating for cellular mechanisms which is lost by p38 deficiency including survival [44,45]. OS treatment in WT cell-containing membranes induced a complex signaling landscape documenting multiple pathways and development of an EMT to ‘mesenchymal state’ phenotype (i.e., clusters 3–6) (Figure 3E,F). Cells from these clusters expressed: (1) pro-EMT transcription factor TWIST (cluster 4 and 5) and ZEB1 (i.e., cluster 6), (2) activation of stress kinases p38 (cluster 3) or JNK (cluster 4), and (3) an increase in mesenchymal markers vimentin (cluster 3) and α-SMA (cluster 3 and 6) and N-cadherin (cluster 3). Various clusters were taken together for data interpretation as spatial-temporal differences are expected in cells on the membrane as they respond to stress signals. Such reconstructed membrane cells are likely still finding their niche to establish themselves and their distinct response to OS. Therefore, collective analysis of clusters was attempted to determine the cell fate of WT cells under OS environment using a cut-off value of 0.6 units from the heatmap as described in the Methods section. In comparison with WT cells, in OS environment, p38 KO cells on the membranes (i.e., cluster 7) expressed an ‘intermediate state’ phenotype showing dominance in epithelial characteristics with only occasional tendency (based on certain markers) to transition. However, cells in cluster 8 under OS environment expressed a baseline ‘epithelial state’ phenotype with characters like that of WT cells under a normal environment (Figure 3E,F). These findings suggest that p38 deficiency in AEC makes them more epithelioid with diminished EMT characteristics. These data guided the next batch of experiments concerning the measurement of specific characteristics of p38 KO cells. Our experimental approaches determined that deficiency in p38 can prevent AEC senescence (based on our prior report and not based on imaging mass cytometry data described above), migration, invasion, and generation of inflammation.

### Confirming senescence and its inhibition in p38 KO cells

**Figure 4:**
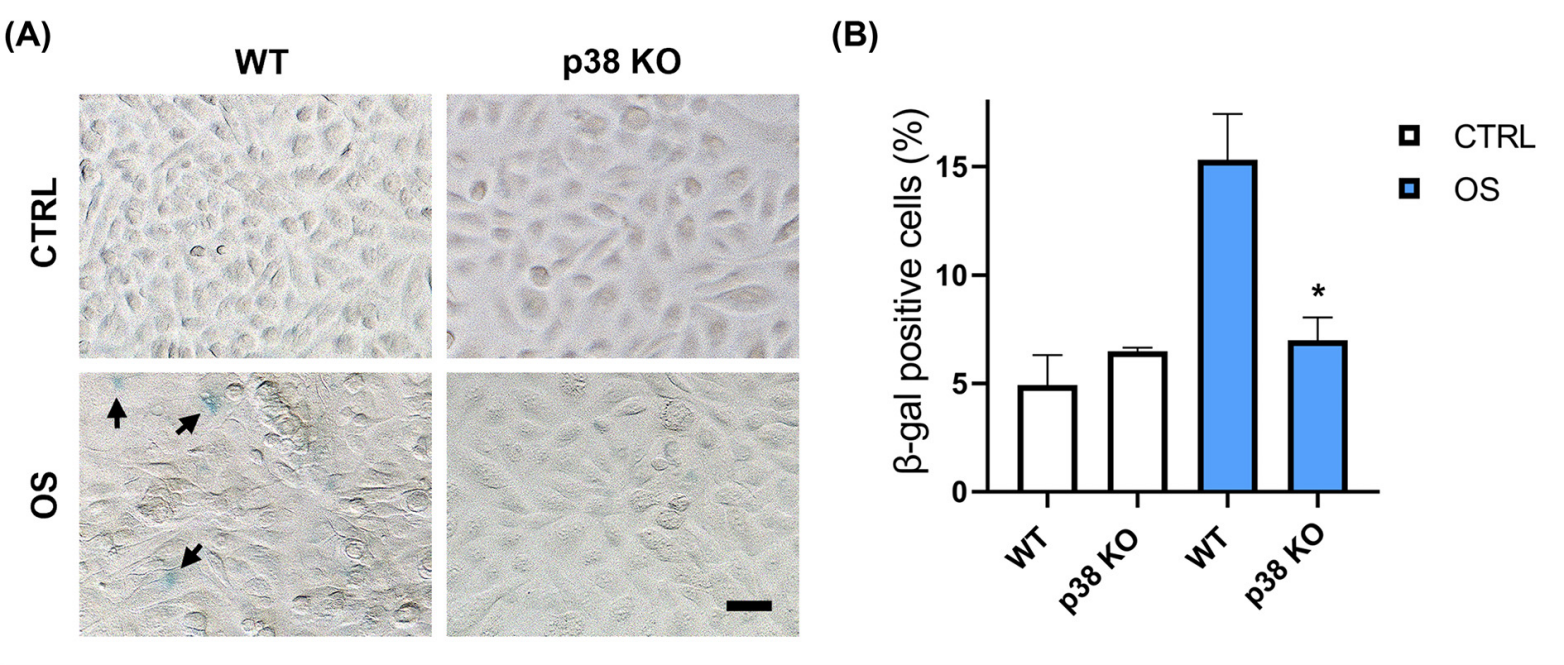
p38 KO AEC show diminished senescence (**A)** Senescence-associated β-galactosidase staining performed at 48 h upon OS treatment comparing WT and p38 KO AEC. Arrows showing senescent cells stained in blue; OS, oxidative stress. Senescence positive cells were quantified and displayed in percentages (**B**); scale bar, 50 µm. The data are presented as the means ± SEM; *P=0.02, Unpaired *t*-test, OS, oxidative stress.

We have already reported that senescence of AEC is one of the key cell fates observed in AEC in response to OS, a condition associated with cellular senescence [11,46]. This phenomenon was decreased when we used p38 inhibitor SB 203580 [25]. We further verified that senescence is also inhibited in AEC after KO of p38. As shown in the representative example of Figure 4A, p38 KO in AEC is associated with significantly decreased SA-δ-Gal staining compared with WT cells in the context of OS. A summary of these data is graphically represented in Figure 4B. This is consistent with what we have reported in other experimental model and further confirms the functional role of p38 in the generation of the senescence phenotype in AEC. It is important to note that not all AEC undergo senescence and senescent cells are unlikely to undergo EMT but remain in the environment to cause localized inflammation and force other cells to undergo senescence [47]. Non-senescent cells can transition under OS conditions. It is unclear which cell will undergo senescence or transition and this fate is likely dependent on multitudes of other factors like the extent of OS induced damage to cell, telomere attrition, and activation of other signaling markers secondary to changes in the cell [23].

### Reconstructed amnion membrane exhibits EMT similar to that observed in an intact amnion membrane in response to OS

**Figure 5:**
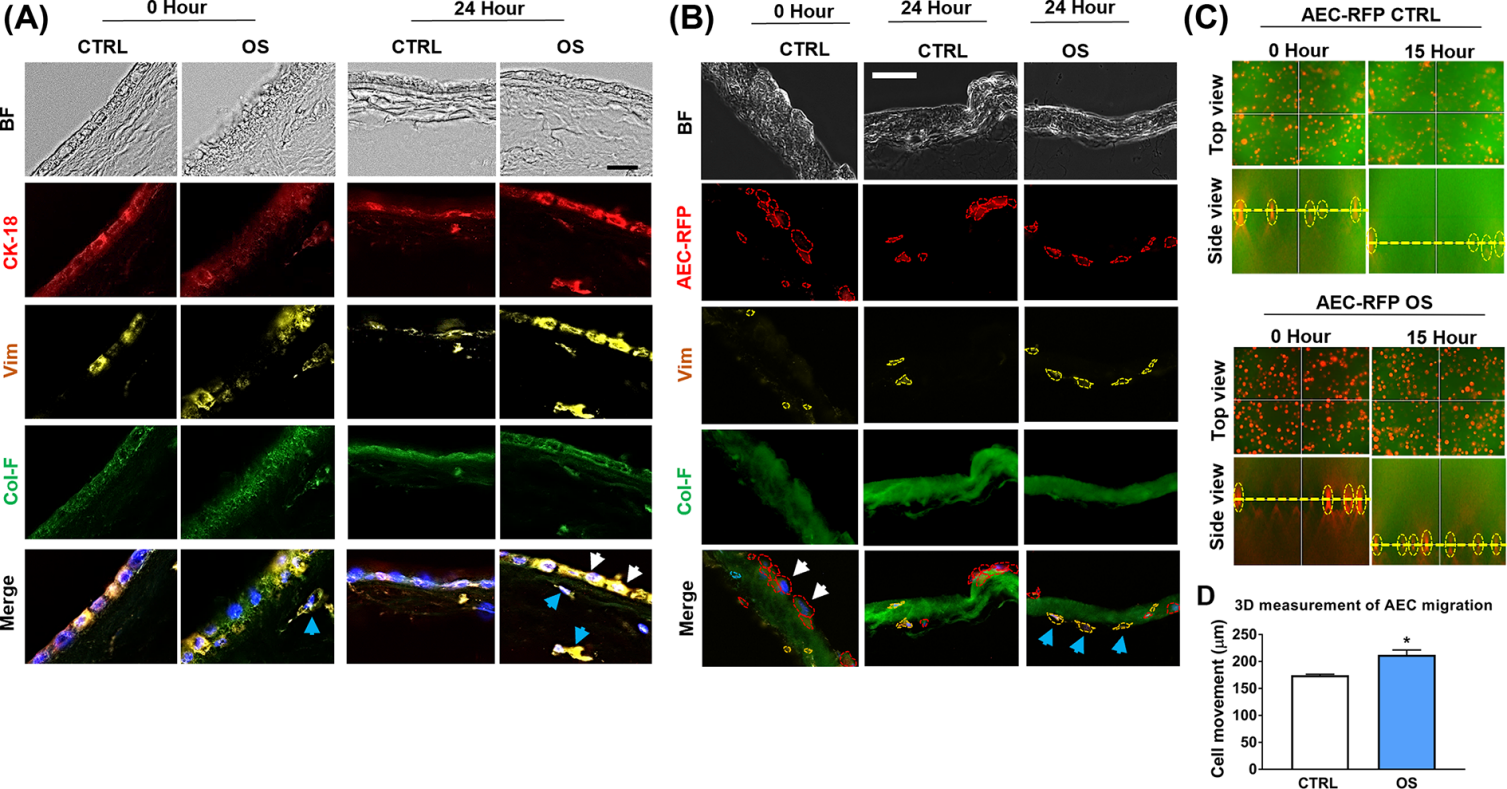
*In vitro* reconstructed amnion membrane mimics the intact amnion membrane for OS-induced EMT (**A**) A representative image of CTRL *vs*. OS-treated intact amnion membrane explant staining of CK-18 (red) for epithelial cell marker and vimentin (yellow) for mesenchymal cell marker. An intact amnion membrane tissue explant from human placenta was stained with green-fluorescent probe Col-F (10 µM) for the visualization of the extracellular matrix (collagen and the elastin probing) using 10 µm thick frozen sections. Twenty-four hours after the OS-treatment vimentin expression was increased in AEC (white arrowhead) and in AMC in the extracellular matrix layer (blue arrowhead) (*N*=3); scale bar: 50 µm; BF, bright field; CK-18, cytokeratin-18; OS, oxidative stress; Vim, vimentin. (**B**) Reconstructed amnion membrane showed a similar vimentin expression pattern to intact amnion membrane against OS. In untreated controls, AEC-RFP cells showed circular epithelial morphology (white arrowhead). Twenty-four hours after OS treatment, AEC-RFP cells showed elongated mesenchymal morphology (blue arrowhead), and the RFP-expressing cells displayed mesenchymal marker vimentin co-expression (blue arrowhead) (*N*=3); scale bar: 50 µm. (**C**) AEC-RFP migrated faster in OS-stimulated condition. 3D display of the CTRL and OS-treated AEC-RFP cells migration through amnion membrane at 0 h and 15 h from Z-stack time-lapse images using Keyence live imaging (*N*=3). (**D**) The graph displays AEC-RFP cells migration in distance (µm) at 15 h which starts from 0 h set-up level using 3D Measure function, BZ-X800 Analyzer software (*N*=3). The data are presented as the means ± SEM; *P=0.01, unpaired *t*-test.

The amnion membranes normally undergo EMT at the end of gestation that is associated with OS-induced p38 activation [19]. To confirm these findings, intact amnion membranes from term not in labor placenta were cultured under normal conditions *ex vivo* in the transwell system (Figure 5A). EMT induction due to OS was confirmed when AEC increased the expression of mesenchymal cytoskeletal marker vimentin compared to untreated controls (Figure 5A, white arrows: AEC, and blue arrows: AMC). These data were consistent with our previous reports [19].

Reconstructed amnion membrane exhibited a similar result as an intact amnion membrane against OS stimuli (Figure 5B). After seeding the AEC-RFP onto a nude membrane, the cells showed classic epithelial circular morphology with low expression of vimentin (Figure 5B, white arrows). OS treatment transitioned substantial number of these cells to elongated vimentin expressing mesenchymal phenotype (Figure 5B, blue arrows). Next, we tracked the migration of AEC-RFP through the nude amnion membrane with Z-stack time-lapse imaging for 15 h under normal and OS environment. 3D measurement of the Z-stack images showed that AEC-RFP cells migrated further (*P*=0.01) through the ECM under an OS environment compared with normal environment (Figure 5C,D).

### EMT and migration is mediated by p38

**Figure 6:**
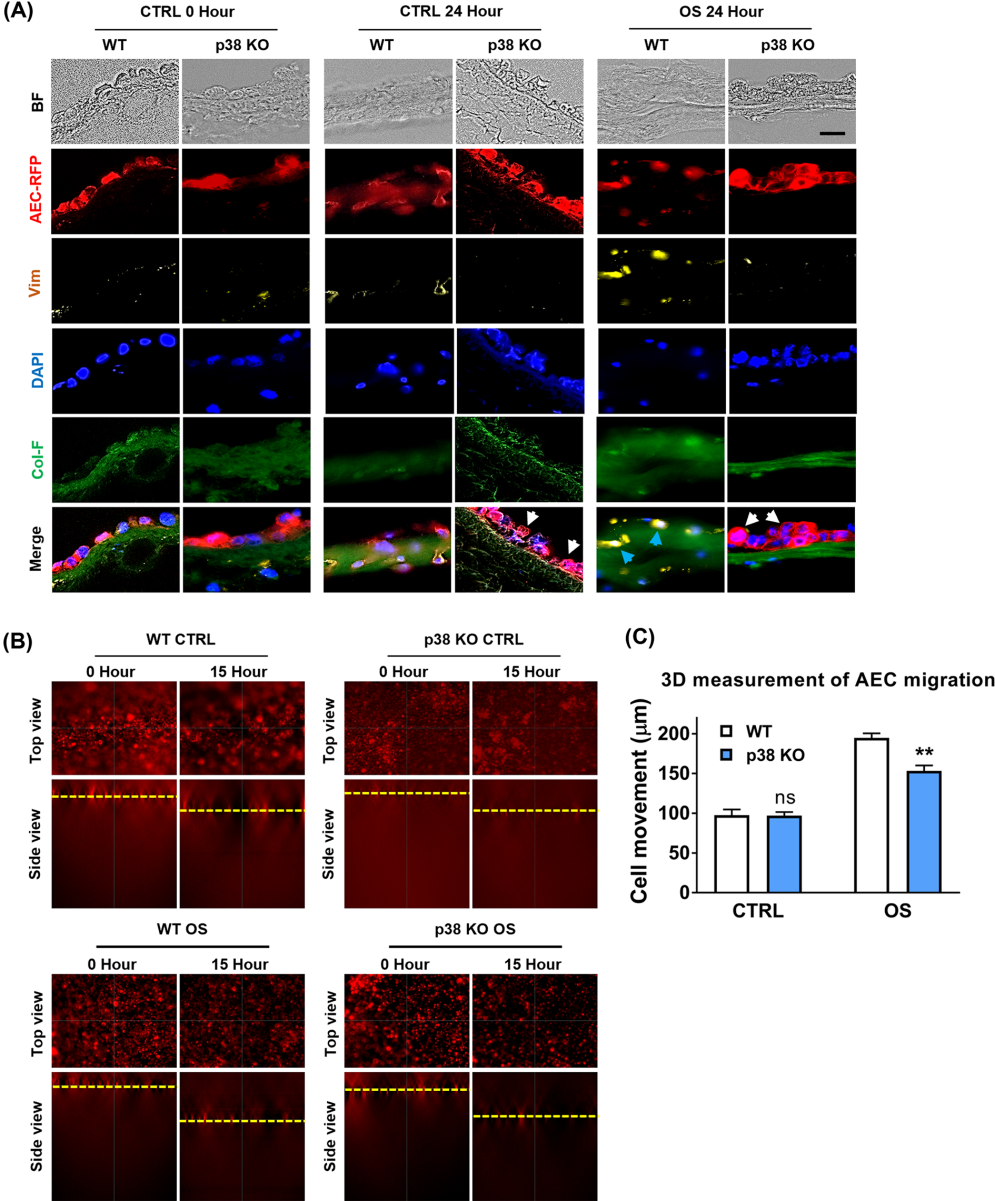
EMT and migration of the AEC are mediated by p38 in the human amnion membrane (**A**) Reconstructed amnion membrane using WT and p38 KO AEC-RFP cells. 120,000 WT and p38 KO AEC-RFP (red) cells were seeded onto Col-F labeled (green) nude membranes in the transwell system. p38 KO AEC-RFP cells morphologically remain unchanged (circular epithelial, white arrows) in both CTRL and OS-treated samples. However, WT AEC-RFP cells morphologically converted to elongated (mesenchymal, blue arrows) and showed higher expression of vimentin 24 h after the OS treatment (*N*=3); scale bar: 50 µm. (**B**) AEC-RFP cells migration is inhibited in the p38 KO cells. 3D display of WT and p38 KO AEC-RFP cells migration through amnion membrane at 0 and 15 h from *Z*-stack time-lapse images using Keyence live imaging (*N*=3). (**C**) The graph displays WT and p38 KO AEC-RFP cells migration in distance (µm) at 15 h which starts from 0 h set-up level using 3D Measure function, BZ-X800 Analyzer software (*N*=3). The data are presented as the means ± SEM; **P=0.001. Multiple comparison two-way analysis of variance (ANOVA).

Next, *in vitro* reconstructed amnion membranes were prepared using WT and p38 KO AEC-RFP to check the involvement of p38 in the EMT and migration processes. OS treatment caused WT AEC-RFP cells to transition as they showed mesenchymal type-elongated morphology (Figure 6A, blue arrows), and these cells expressed mesenchymal marker vimentin (Figure 6A, blue arrows). Interestingly, these cytoskeletal changes were not observed in p38 KO AEC-RFP cells, and these cells remained morphologically uniform in the amnion membrane (Figure 6A, white arrows). These results confirmed our imaging mass cytometry analysis results (Figure 3F) suggesting that p38 KO cells remained relatively epithelioid after a prolonged period of OS treatment (Figure 6A). Next, we conducted a time-lapse analysis on the reconstructed amnion membrane to track the migration of WT and p38 KO AEC-RFP cells. The migration distance of the p38 KO AEC-RFP cells was significantly lower (*P*=0.001) compared with that of WT cells (Figure 6B,C). These data suggest that OS-induced EMT and migration of the AEC in the amnion membrane is mediated by p38.

### TGF-β-induced EMT is blocked by p38 KO

**Figure 7:**
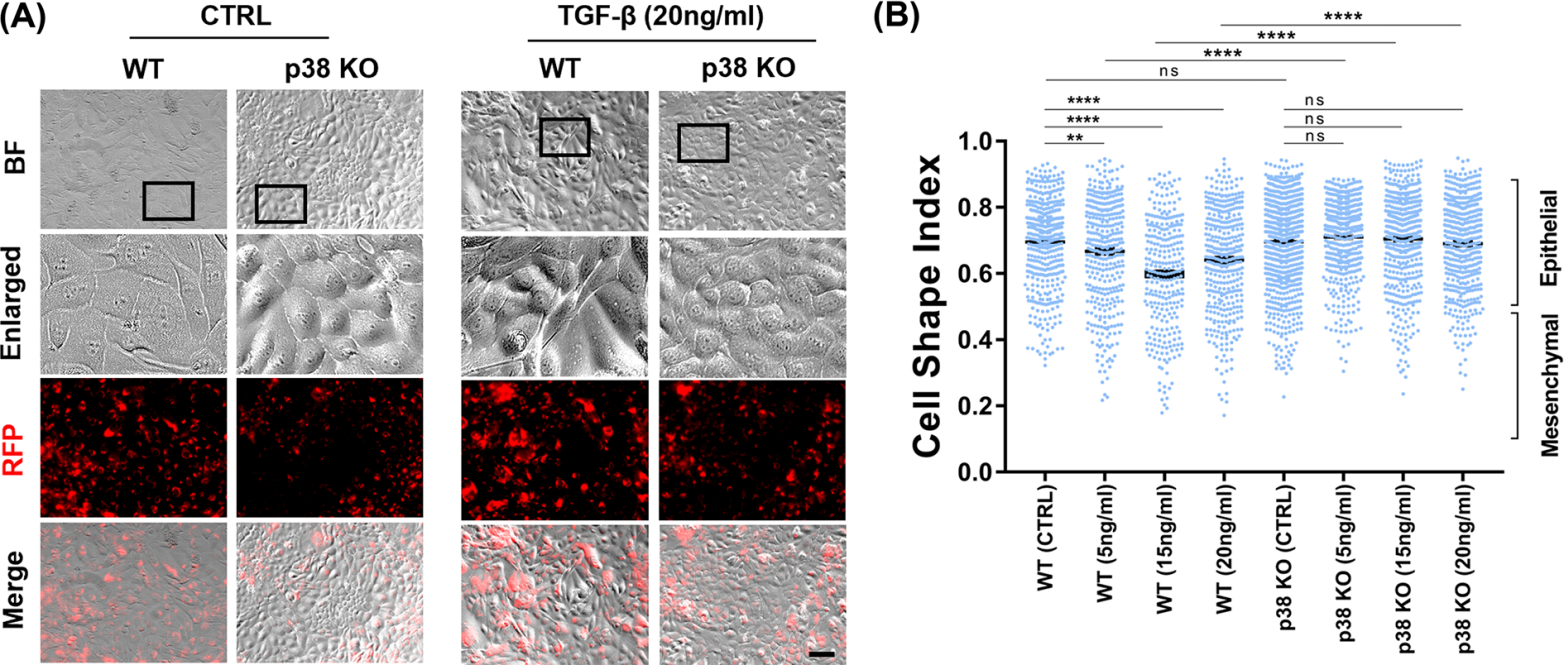
The loss of p38 blocked TGF-β-induced EMT in cell morphology (**A**) p38 KO AEC-RFP cells lacked TGF-β-induced EMT morphological changes. WT and p38 KO AEC-RFP cells were treated with EMT inducer TGF-β at 5, 15, and 20 ng/ml concentrations for 7 days. Representative bright field images displaying epithelial cells’ reaction against TGF-β treatment in morphology at day 7 (*N*=3); scale bar: 100 µm. (**B**) Cell morphology analysis comparing WT and p38 KO AEC-RFP cells at day 7 upon TGF-β treatments. Cell shape analysis was performed using ImageJ software (*N*=3). The data are presented as the means ± SEM; **P<0.01, ***P<0.001, ****P<0.0001**.** Multiple comparison one-way analysis of variance (ANOVA). BF, bright field; RFP, red fluorescent protein; ns, not significant.

To confirm that p38 is the mediator of EMT in AEC and the observed data are not specific to the type of OS inducing reagent (cigarette smoke extract), WT and p38 KO AEC-RFP cells were treated with three different doses (5, 15, and 20mng/ml) of EMT inducer TGF-β [19,48,49] for up to 10 days. As we have reported earlier and mentioned above [18] TGF-b auto-phosphorylates p38 in AEC and therefore the signaling mechanisms are interlinked. Seven days after the initial treatment of TGF-β, WT cells showed a mesenchymal fibroblastoid morphology (Figure 7A) along with a lower cell shape index (CSI) (closer to 0.5) confirming EMT (Figure 7B). This phenotypic change was observed regardless of TGF-b doses used compared with untreated WT cells (Figure 7B). However, p38 KO cells maintained an epithelial morphology and maintained a high CSI (closer to 1 indicative of epithelial shape) regardless of TGF-β treatment (Figure 7A,B). Morphological analysis was also performed in the WT and p38 KO AEC-RFP cells after 10 days of TGF-β treatment (Supplementary Figure S4). The morphological changes of WT cells were dose-dependent; however, p38 KO cells remained unchanged (Supplementary Figure S4). Taken together, TGF-β-induced EMT in AEC were also blocked when p38 is absent in the AEC. This further confirms the role of p38 in this process.

### p38 KO reduces the inflammation

**Figure 8:**
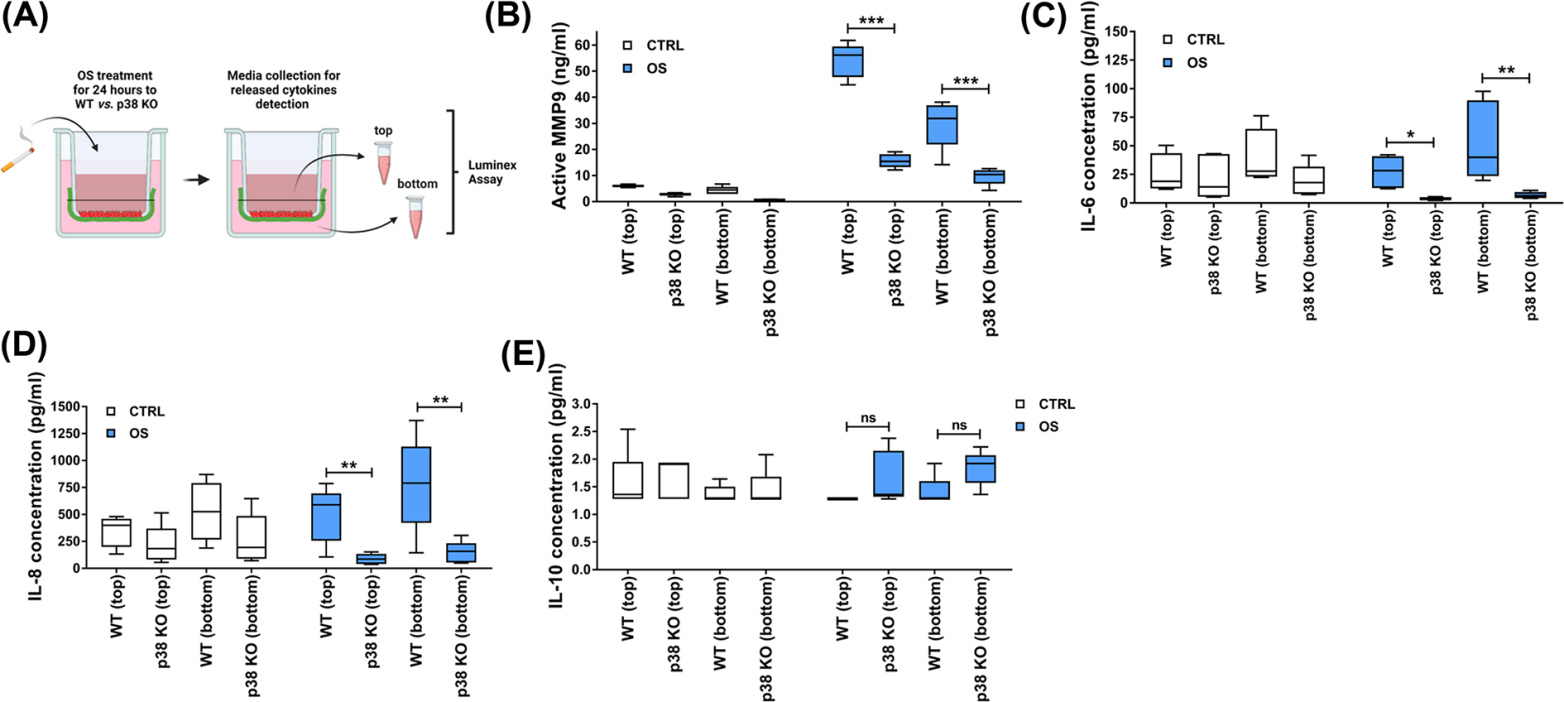
OS-induced inflammation is inhibited in p38 KO cells (**A**) Cartoon illustration of media collection from reconstructed WT and p38 KO amnion membrane in the transwell system. Media from top side of the transwell represents the cytokines released from epithelial cells, while the media from bottom side of the transwell represents the cytokines from mesenchymal cells. Released inflammatory cytokine levels was detected 24 h after the OS-treatment (**B–E**), *N*=5. The data are presented as the means ± SEM; *P<0.05, **P<0.01, ***P<0.001. Multiple comparison two-way analysis of variance (ANOVA); ns, not significant.

Senescence and EMT generate inflammation, and this can facilitate cellular migration toward the matrix that requires involve degradation of the basement membrane and other ECM collagens. This localized inflammation is a classic sign of both senescence and EMT, where in many instances, inflammation helps tissue remodeling [50–54]. To check the involvement of the p38 in OS-induced inflammation, culture media were collected from the top (AEC) and bottom (migrated cells) of the transwell chambers of reconstructed WT and p38 KO AEC-RFP-containing amnion membranes upon OS treatment (Figure 8A). Without OS (white bars), media from WT and p38 KO cell-containing reconstructed amnion membrane contained baseline levels of MMP-9, IL-6, IL-8, and IL-10 (Figure 8B–E). After exposure to OS, media from both top and bottom chambers of the WT cell-containing membrane showed significantly higher concentrations of active MMP9 and inflammatory cytokines IL-6 and IL-8 compared with p38 KO cell-containing amnion membranes (Figure 8B–D); however, IL-10 showed no significant difference (Figure 8E). These results determined that p38 induced changes to AECs can also regulate local inflammation. To further validate p38’s role in the development of inflammatory processes, next a murine model of pregnancy was utilized.

### p38 cKO mice exhibit reduced senescence and inflammation in the amniotic sac

**Figure 9:**
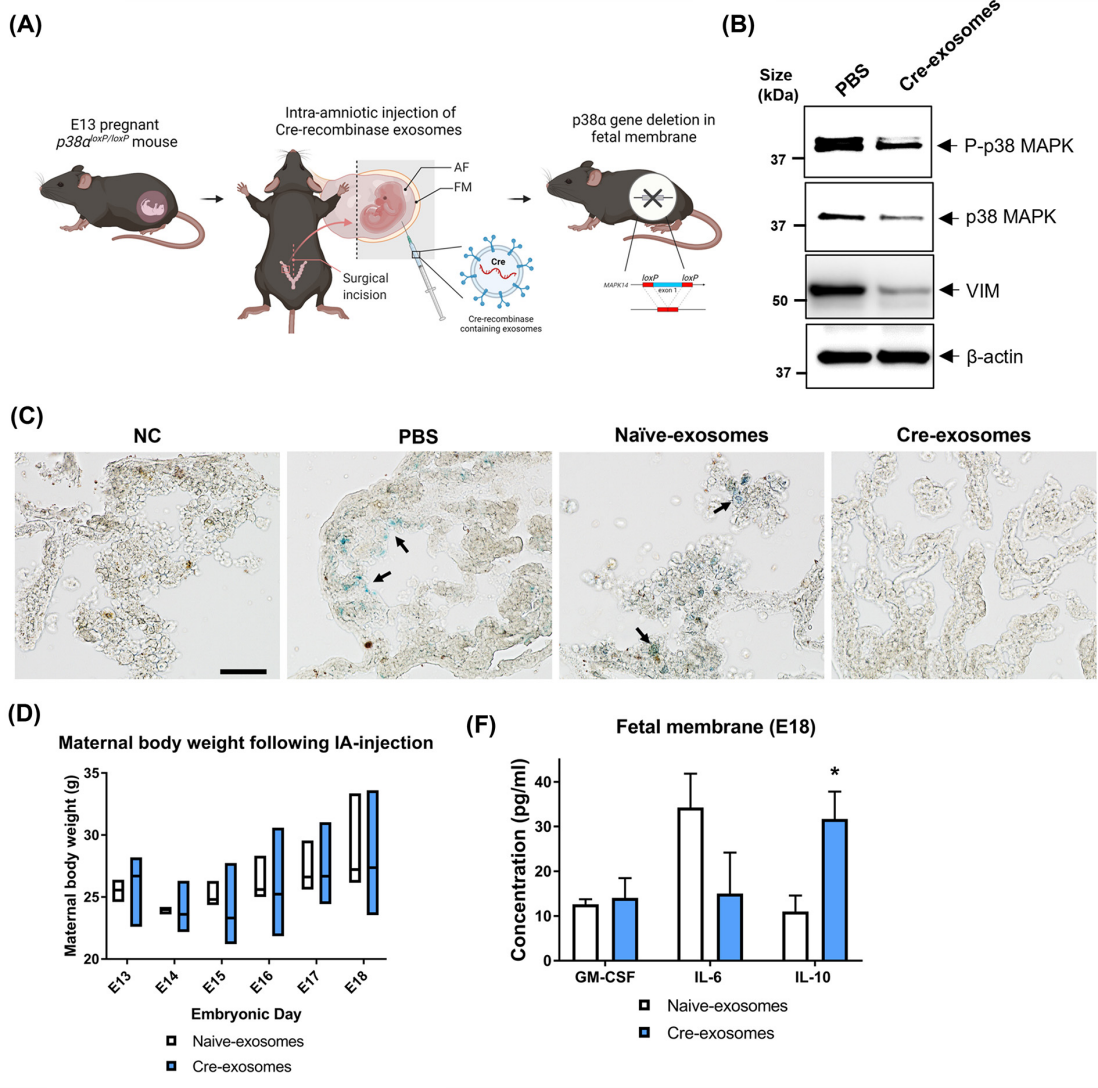
p38 cKO in fetal membrane exhibited reduced senescence and inflammation in mice (**A**) Schematic of conditionally knocking out p38 in fetal membrane using *p38α^loxP/loxP^* mice and Cre-recombinase containing exosomes. E13 pregnant mice were exposed to Cre-recombinase intra-amniotically for cKO of p38 in fetal membranes; AF, amniotic fluid; FM, fetal membrane. (**B**) Western blot analysis confirming the deletion efficiency of p38 in the fetal membrane lysates (20 µg of total protein applied) 30 h after the Cre-exosomes (1.00E+10 per amniotic sac) injection to E13 mice. Vimentin was used to determine EMT. β-Actin was used as a loading control (*N*=3). (**C**) A representative image of senescence staining using 10 µm thick frozen tissue sections from mice fetal membrane. Samples were collected 30 h after the Cre-exosomes (1.00E+10 per amniotic sac) injection to E13 mice (*N*=3). As controls, PBS, and naïve exosomes (1.00E+10 per amniotic sac) were injected; scale bar: 100 µm; NC, negative control. (**D**) Maternal body weight throughout gestation (E13-E18) after naïve- and Cre-exosomes (both 6.75E+08 exosomes per amniotic sac) injection (*N*=4). The data are presented as the means ± SEM. (**E**) Inflammatory cytokines in the fetal membrane tissue lysates at E18 performed with multiplexed ELISA (*N*=4). The data are presented as the means ± SEM; *P=0.04. Multiple comparison two-way analysis of variance (ANOVA).

Immune homeostasis and inflammatory balance during gestation are essential for the maintenance of pregnancy. Increased activation of p38 at term in response to OS leads to EMT, cellular senescence, and inflammation that imbalances immune homeostasis and contribute to labor onset; thus, making pregnancy a model system to study p38’s role *in vivo*. Since p38 null mice are embryonically lethal [27], *p38α^loxP/loxP^* mice (Supplementary Figure S5A) were used to conditionally knockout the p38 in the fetal membrane by exposing it with Cre-recombinase (Cre) containing exosomes (Supplementary Figure S5B,C) to study the role of p38 in senescence, EMT, and inflammation *in vivo* (Figure 9A). The generation and use of Cre exosomes have been reported previously by our laboratory [38]. Before conducting the intra-amniotic injection in *p38α^loxP/loxP^* mice for p38 cKO experiments, the effectiveness of Cre-exosomes was validated using TdTomato reporter mice (Supplementary Figure S5D). As a control, either PBS or naïve-exosomes (same number of exosomes to Cre-exosomes) were injected. Thirty hours after the exposure to Cre-exosomes, the TdTomato loxP site in the fetal membrane was deleted enabling the activation of eGFP expression (Supplementary Figure S5D). Upon confirmation of the Cre functions in exosomes, E13 pregnant *p38α^loxP/loxP^* mice were injected intra-amniotically to delete p38 conditionally in the fetal membrane (Figure 9A). E13 pregnant mice were chosen to conditionally KO the p38 in the fetal membrane based on previous mouse experiments that showed natural gestational senescence in the fetal membranes starting at E14 [40]. Figure 9B confirms the conditional deletion of p38 in the fetal membrane. To validate EMT caused by p38 deletion in the p38 cKO fetal membranes, vimentin expression was examined. As shown in Figure 9B, the vimentin expression was reduced in the p38 cKO samples compared with controls. In addition, the loss of p38 in the fetal membrane reduced senescence in the fetal membrane that may also explain the drop in localized inflammation (Figure 9C, black arrows: senescense cells).

EMT and senescence induced by p38 are associated with inflammation as seen in our* in vitro* experiments described above. Therefore, we first checked if Cre injection has any impact on the maternal side, and subsequently determined the inflammatory cytokine levels in the mouse fetal membranes. Injection of either Cre or naïve exosomes did not affect maternal body weight suggesting that pregnancy was progressing normally in this model (Figure 9D). Examination of gestational inflammatory cytokines in the fetal membrane showed a significant increase in IL-10 levels in Cre-injected animals along with no change in proinflammatory GM-CSF and IL-6 concentrations (Figure 9E). It is interesting to note that IL-10 cytokine level was elevated during normal gestation in the absence of p38; however, remained unchanged with OS-stimulated case (Figure 8E). These data further support the hypothesis that p38 may control local inflammation either directly or by regulating senescence and EMT. We did not check the impact of p38 in controlling NF-kB in our mouse models, although there are several reports that p38 can directly regulate NF-kB and thus control inflammation [55–58].

## Discussion

Stress signaling activated cellular derangements are associated with various pathologies including the development of tumors [4,59] as well as pregnancy complications such as fetal membrane rupture and preterm birth [15,23]. In the present study, we developed a model to determine cell fate in response to OS-associated processes that activate stress signaler p38 activation. To test the impact of p38 activation, we developed a cell-free extracellular matrix region derived from the human amnion membranes that are otherwise layered with AEC. The addition of isolated AEC and their subsequent exposure to OS caused cellular senescence, EMT, cell migration, and invasion into the ECM causing matrix degradation and inflammation, an effect that was reversed when AEC were devoid of p38. These data were functionally validated when a conditional KO of p38 in fetal membranes in mouse models of pregnancy showed a reduction in EMT and inflammation. To test the properties of p38, we used CRISPR/Cas9 KO cells and conditional KO in animals were created using exosomes engineered to contain Cyclic recombinase that was administered intra-amniotically to deliver them at the site. These changes in mouse fetal membranes were also associated with anti-inflammatory response in the fetal membranes where IL-10 concentration was increased in the absence of p38. Reduction in IL-10 in the intrauterine environment has been implicated as one of the inflammatory mechanisms associated with PTB and pPROM [60–62]. These data suggest that p38 may mechanistically regulate local IL-10 action via cellular derangements.

Activation of p38 can be a cause of concern in various pathological conditions. Chronic inflammatory conditions like rheumatic arthritis, inflammatory bowel disease, aging-related bone disorders, and allergic asthma are a few examples of p38 associated pathologies [63–67]. Similarly, breast carcinoma growth and its metastatic capacities are enhanced by p38 signaling [68]. As reported, OS-associated preterm birth and premature fetal membrane ruptures can also result from untimely and or prolonged p38 activation. Oxidative stress-induced excessive activation of p38 forces accumulation of proinflammatory mesenchymal stromal cells in the ECM that can weaken the membranes by causing localized inflammation and damage. MMP9 activity is decreased in the absence of p38, and it can also minimize Type IV basement collagen degradation, the connector of AEC to the ECM layer (Figure 8B).

Phosphorylation of p38 MAPK is requirement for p38 MAPK activity and a marker for p38 MAPK activity. However, sustained p38 MAPK kinase activity can cause cellular senescence, EMT, and inflammation. To note, these types of cell fates have a distinct physiological or pathological manifestation in different cell types under distinct environments. Therefore, the mechanisms and duration of p38 activation are tightly regulated in a cell [69]. A well-reported p38MAPK activation process in most cells under cellular stress conditions is via ROS-sensitive thioredoxin-apoptosis signal-regulating kinase (Trx-ASK1)-signalosome, which activates the p38 stress response signaling cascade. In our previous study, we have already shown that this canonical pathway does not exist in human AEC [70,71]. In systems in which cellular proliferation is preferred in topographically defined regions (as seen in early stages of pregnancy), p38 activation is regulated non canonically. In our model, p38 activation is regulated by ROS-induced TGF-b and its receptor-mediated signaling cascade; hence, the functional properties are rather different at distinct phases of pregnancy due to changes in the oxidative environment. Amniotic fluid TGF-b levels are also regulated by an intrauterine oxidative environment that changes during pregnancy. Hypoxic first and third trimesters and hyperoxic second-trimester environments are regulators of TGF-b concentrations in the amniotic fluid and in turn regulate p38 activation in cells. TGF-β via TGF-b receptor-TGF-β-activated kinase1 and TGF-β-1-binding protein1 signaling pathway activates p38. Regulated p38 favors cell cycle progression and growth and an enhanced growth is seen in second in response to OS type situation *in utero*. TGF-b is a known activator of EMT in AEC [19] and mediates remodeling. Here, we showed that OS-induced senescence and EMT in AEC are regulated by p38 and lack of p38 in cells prevents their migration and inflammation.

MAPK pathways are interlinked, and their network consists of cross-talks and compensatory pathways implicated in stress signaling [72,73]. These overlapping functions and compensation for the loss of one molecule often provide challenges in controlling cell fate-associated pathologies. Cross-talks between other MAPKs (extracellular signal-regulated kinases (ERKs) 1 and 2 (ERK 1/2), c-Jun amino-terminal kinases, or stress-activated protein kinases (JNK/SAPK)1-3 [74], may compensate for p38's function. Although ERK/JNK have other cellular biological functions besides complementing p38 function, the lack of their compensatory functions was evident in our system when cells stopped transition and migration in the absence of p38. p53, an antitumor cell cycle regulator and proapoptotic and senescence molecule, implicated in decidual senescence and associated with preterm birth pathology [46,75] is also not compensating for the loss of p38 [76]. Exclusive functions performed by various MAPKs in cell fate affecting roles reported in KO animal models also further validates our data [77].

The sustained overexpression of the p38 is a characteristic of chronic inflammation in aging tissues and promotes multiple human diseases. Therefore, p38 can be an ideal therapeutic target for reducing deleterious changes to the cell and its inflammatory environment [78,79]. Several therapeutic approaches have been designed to reduce the activation of upstream mediators and downstream effectors of p38 pathways [80,81]. Besides, therapeutic strategies to directly impact p38 functions have also been designed [9]. During pregnancy, inhibiting p38 function is not an option as its activity is needed for cell cycle progression and tissue growth. A better approach is to reduce excessive p38 activation and function to minimize the impacts on cellular changes described above. OS-induced damages to fetal membrane cells are the key trigger for excessive p38 during pregnancy. Excessive p38 activation is difficult to determine in intrauterine organs until clinical manifestations such as early labor or membrane rupture occur. Therefore, minimizing damage should involve risk assessment and activation of ROS-induced damages.

There are a few limitations that still needs to be addressed in the future. The numbers of cells migrated are fewer in our experimental model to determine the activation of multiple pathways. Although our imaging mass cytometry analysis determined EMT and inflammation supporting our prior data and hypothesis of p38’s role in these processes; it is likely that other migration and invasion-related markers could have been determined if a larger number of migrating cells had been examined. This limitation is related to the size of the membrane section which can be fit in a trans-well as shown in Supplementary Figure S1 (step 4). Inclusion of more markers could determine additional pathways with an expanded version of the current models. Cre exosomes were beneficial in our studies to create a knockdown of p38, but this effect is not fetal membrane specific as the destination of exosomes could be anywhere and the effect can be a non-target manner. This prevented us from determining the phenotypic outcome in these models (e.g. delay in preterm birth in p38 knockdown models) as the effect in other fetal and maternal tissues may confound the data.

**Figure 10:**
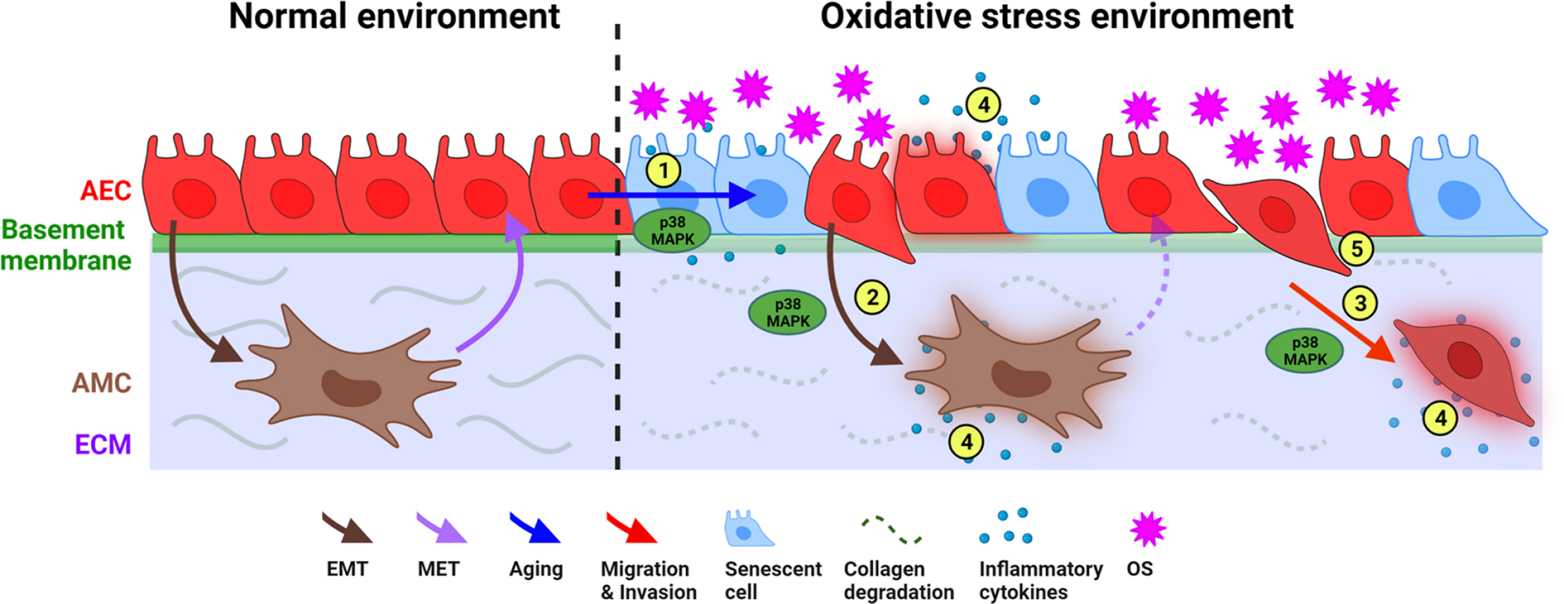
Schematic summary of p38 role in amnion membrane homeostasis The amnion layer of the fetal membrane consists of AEC (red) and AMC (brown) in collagen-rich ECM (purple). Upon exposure to oxidative stress-inducing exposomes, the characteristics of AEC change to several different paths involving p38 activation in the amnion membrane: (1) Senescence of AEC, (2) EMT of AEC, (3) migration of AEC through ECM, (4) senescence and EMT cause localized increases in inflammatory cytokines, and (5) production of active-matrix metalloproteinase (MMP9) by senescent and transitioning AEC degrade the basement membrane and other collagen types in ECM; AEC, amnion epithelial cells; AMC, amnion mesenchymal cells; ECM, extracellular matrix; EMT, epithelial–mesenchymal transition; MET, mesenchymal–epithelial transition; OS, oxidative stress.

Stress signaler p38 can determine the fate of cells and the tissue function in multiple ways. In the present study, we used AEC that use the controlled function of p38 during pregnancy to remodel the tissue through a cyclic transition process (Figure 10). This is a physiologic need during pregnancy for fetal growth and pregnancy maintenance. On the contrary, overactivation of p38 in response to increased OS response in the uterine cavity can cause senescence and a terminal state of EMT (where MET power of cell is diminished). Both senescence and EMT create an unstable tissue environment with inflammation and matrix degradation beyond remodeling or repair capabilities. This state of the amnion membrane makes it dysfunctional and no longer capable of supporting pregnancy. This also is a physiologic need for delivery of the fetus at term; however, pathologic over activation of p38 in response to an exposome exposure by the mother during pregnancy can cause premature rupture of the membrane and preterm birth. This phenomenon and function of p38 may not be restricted to fetal membranes and pregnancy. In our model, no other stress signalers compensated for the loss of p38 suggest that p38 can be a sole contributor to cell fate under specific oxidative and endocrine environments. This is a function that can occur in various tissue environments where p38-mediated cellular changes can promote tumor growth and metastatic cellular invasions. How p38 functions can be balanced and regulated may help to understand the metastatic potential of cells in certain tumors. Therefore, the present study demonstrates p38’s capacity contributing to tissue remodeling as well as tissue destructions under specific environments, where we provide a functional model to study p38.

## Clinical perspectives

- Clinical perspective Stress signaler p38MAPK activation is hypothesized to cause these pathologic events leading to human fetal membrane rupture and preterm birth. However, the precise mechanistic role of p38 and its contributions to these conditions is still unclear. To determine the mechanistic role of p38MAPK in these pathologies, we used multiple molecular and cellular biological approaches and determined that p38 MAPK impacts the normal functioning of fetal membrane cells under oxidative stress conditions. Oxidative stress due to various risk factors is common during pregnancy and p38MAPK activation can be detrimental. Here in we report the role of p38MAPK in causing cell fate in human amnion membrane and future trials can address the mechanisms to curtail its premature activation to reduce the risk associated with membrane dysfunction initiated by p38MAPK overactivation. A brief summary of the results.
- The human amnion membrane undergoes physiologic senescence and EMT during normal pregnancy to maintain tissue homeostasis and this process is facilitated by p38MAPK. This is a requirement for the maintenance of pregnancy and to promote parturition. In this report, we show that knocking down p38MAPK from the membrane cells *in vitro* as well as its conditional KO from mouse fetal membranes mechanistically proved its impact on fetal membrane senescence and EMT. Our study suggested that overwhelming activation of p38 in response to OS-inducing risk exposures can have an adverse impact on cells, cause cell invasion, inflammation, and ECM degradation detrimental to tissue homeostasis. Point 3. The potential significance of the results to human health and disease.
- Understanding the mechanistic mediators of human fetal membrane rupture and preterm birth will enable the development of strategies to prevent untimely activation of such factors from causing pathologic outcomes in pregnancies. We expect that p38MAPK can be a target to delay the premature aging of fetal membranes that will prevent fetal membrane dysfunction

## Code Availability

No unpublished code was used in this manuscript.

## Supplementary Material

Supplementary Figures S1-S5 and Table S1

## Data Availability

All data generated or analyzed during this study are included in this article. The datasets generated during and/or analyzed during the current study are available from the corresponding author (Ramkumar Menon) on reasonable request.

## Abbreviations

AEC: amnion epithelial cells
BF: bright field
ECM: extracellular matrix
EMT: epithelial–mesenchymal transition
ERK: extracellular signal-regulated kinase
KO: knockout
MAPK: mitogen-activated kinase
MET: mesenchymal–epithelial transition
OS: oxidative stress
RFP: red fluorescent protein
TGF: transforming growth factor
