# Supplementary material for: Stress signaler p38 mitogen-activated kinase activation: a cause for concern?
Source: Clin Sci (Lond). 2022 Nov 14;136(22):1591–614. doi: 10.1042/CS20220491 (PMC9664350; doi:10.1042/CS20220491)
Supplement: Supplementary Figures S1-S5 and Table S1 [file CS-2022-0491_supp.pdf]

Supplementary Information

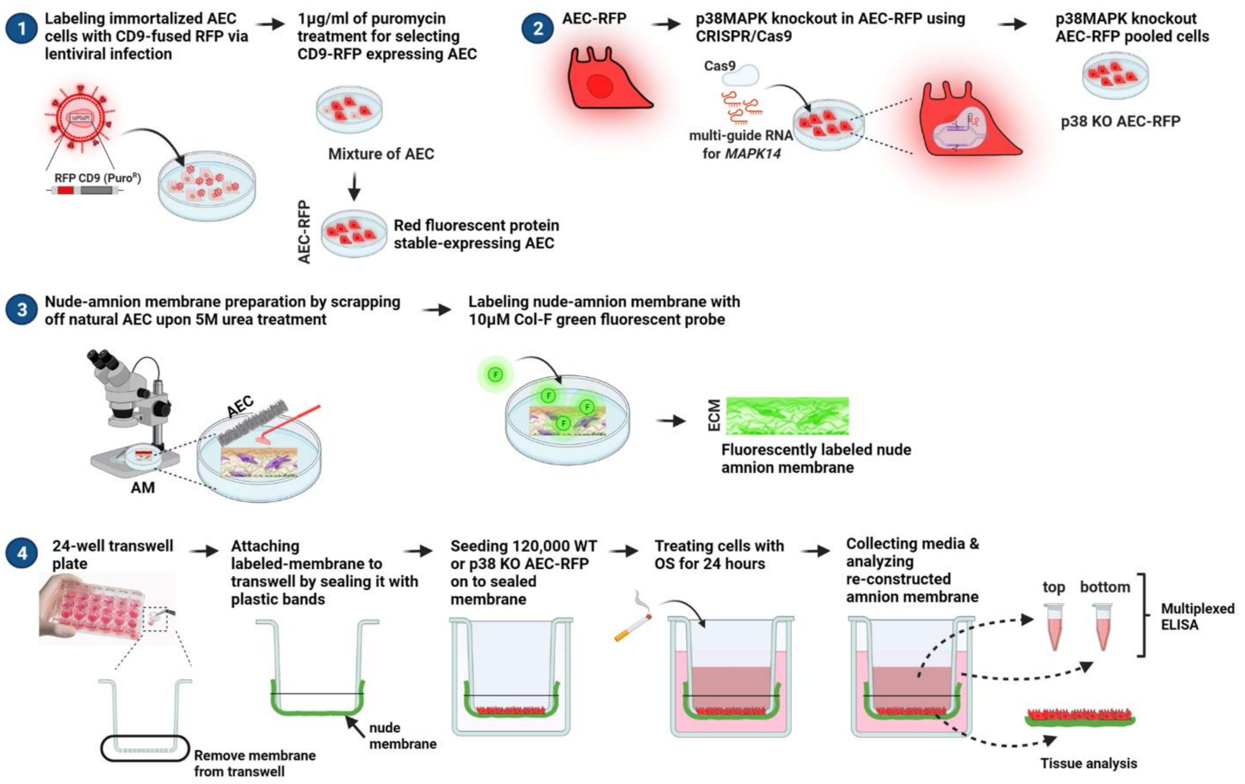

Figure S1. Experimental workflow of p38 knockout (KO) AEC-containing amnion membrane (AM) model system. 1: Labeling of AEC with RFP. 2: Knocking out of p38 in AEC-RFP using CRISPR/Cas9 system. 3: Preparation and probing of nude AM. 4: Reconstructing AM using WT and p38 KO AEC-RFP cells using a trans-well system. AEC, amnion epithelial cells. RFP, red fluorescent protein. KO, knockout. AM, amnion membrane. ECM, extracellular matrix. OS, oxidative stress.

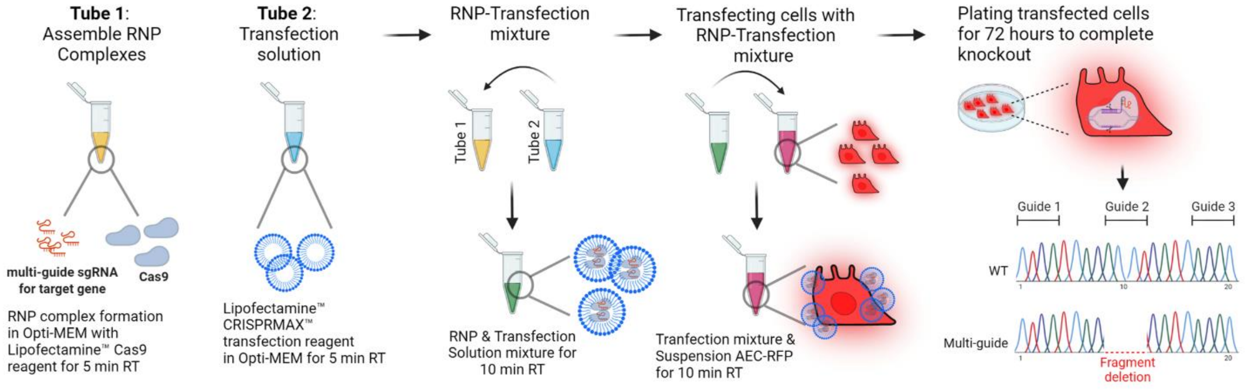

Figure S2. Schematic workflow of CRISPR/Cas9-based knockout (KO) cell line generation using multi-guide sgRNAs co-transfection. RNP, ribonucleoprotein. sgRNA, single guide RNA. RT, room temperature.

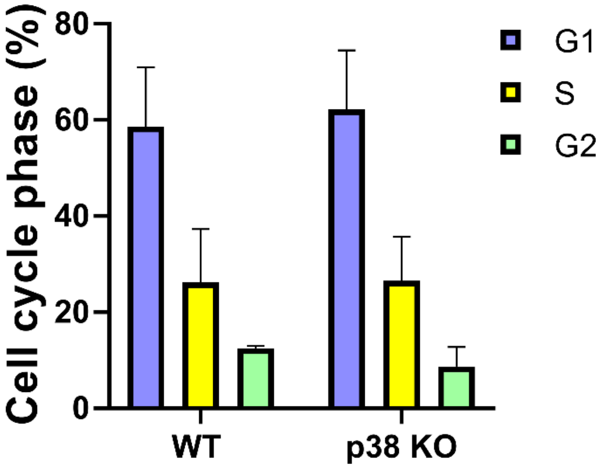

Figure S3. Cell cycle analysis of WT and p38 KO cells under control condition. Cell distribution quantification for each phase of the cell cycle was performed from at least 10,000 cells per sample. Comparison analysis between WT and p38 KO cells show similar results for each cell cycle-phase (G1: P= 0.846, S: P= 0.982, G2: P= 0.212). Data represented as mean ± SEM (N=3). Unpaired t-test.

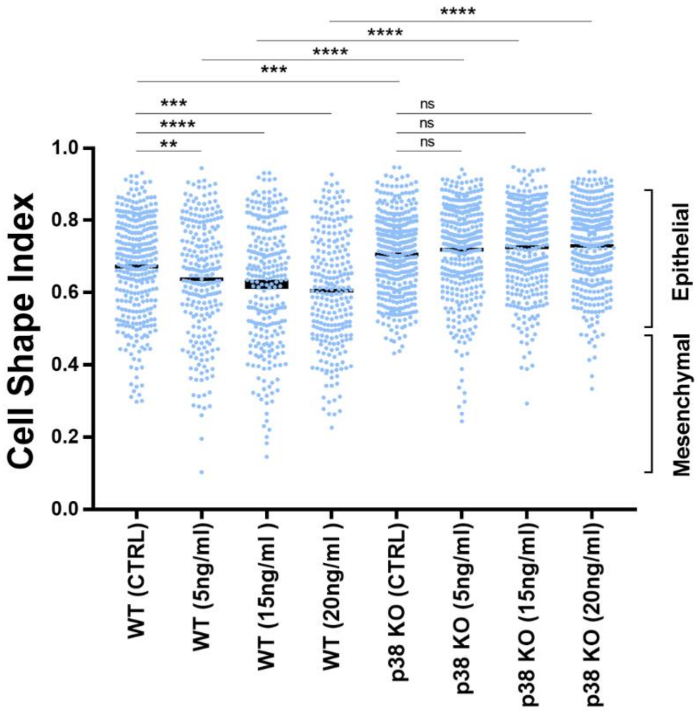

Figure S4. TGF-β-induced EMT in cell morphology at day 10. Cell morphology analysis comparing WT and p38 KO AEC-RFP cells 10 days after 5 ng/ml, 15 ng/ml and 20 ng/ml of TGF-β treatments. CSI analysis was performed using Image J software (N=3). The data are presented as the means ± SEM. *P<0.05, **P < 0.01, ***P <0. 001. Multiple comparison one-way analysis of variance (ANOVA). ns, not significant.

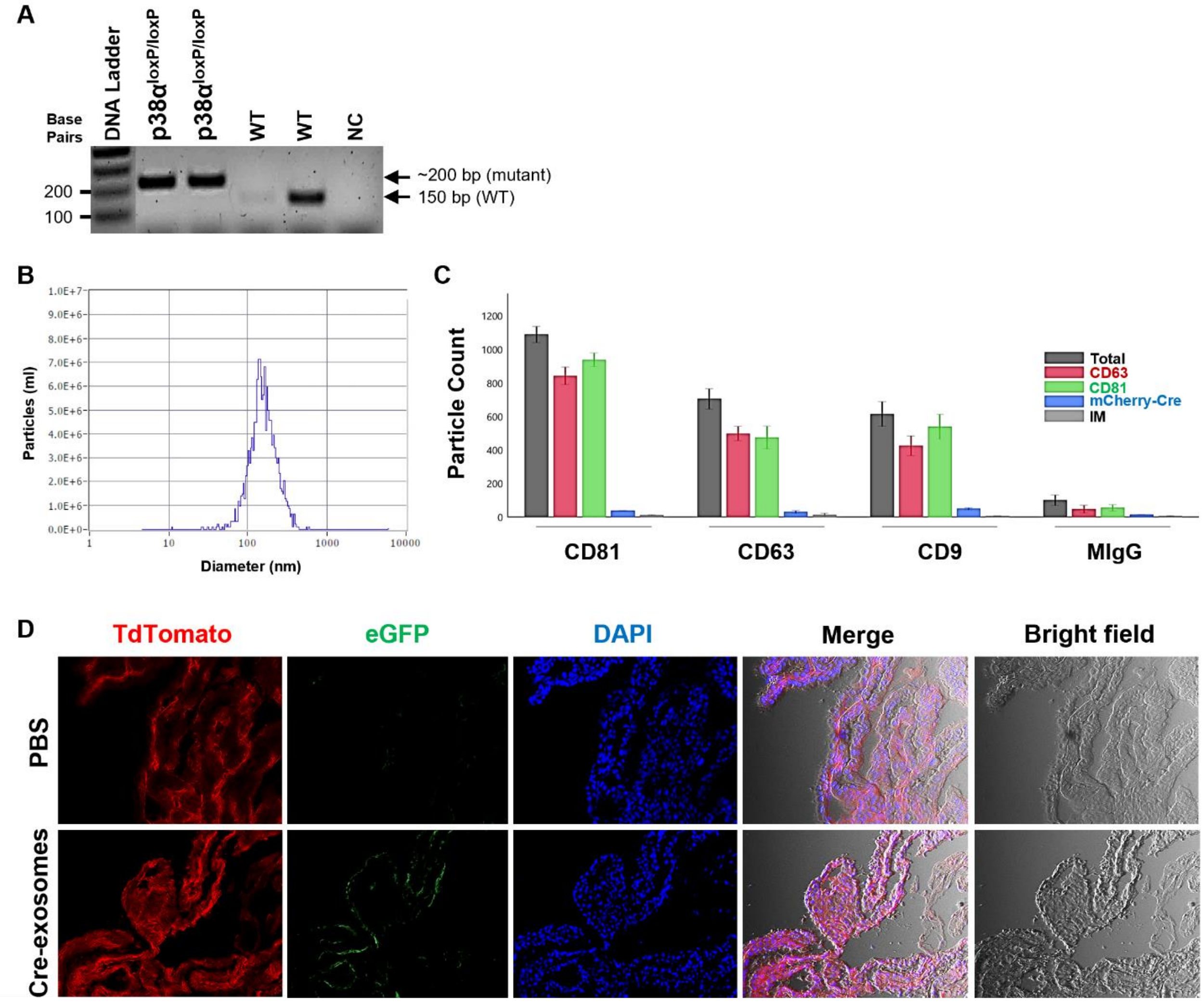

Figure S5. p38 cKO mice preparation. (A) p38αloxP/loxP mice validation with genotyping. PCR was conducted using tail snip from 3 weeks old mice. Agarose gel electrophoresis images showing MAPK14 mutant PCR product (~200bp) which was detected in p38αloxP/loxP mice. WT PCR product (150 bp) is showing as a WT controls. NC, negative control. **(B)** Cre-recombinase containing exosomes validation with a ZetaView analysis for exosomal size distribution, and ExoView analysis for CD63, CD81 tetraspanin markers, and mCherry-fused Cre-recombinase cargo detection **(C)**. **(D)** Cre-exosomes efficiency validation with TdTomato reporter mice in fetal membrane. Cre-recombinase containing exosomes treated fetal membrane showing eGFP

expression confirming the deletion of loxP flanked TdTomato insertion. 10 µm thick frozen fetal membrane tissue samples were stained with DAPI (nuclear detection) and visualized for TdTomato and eGFP expressions.

Numbers	Antibodies	Metals	Source	Cat. No.	Concentration used (µg/ml)	Markers	
1	RFP	146Nd	Novus	NBP1-69962	0.5 µg/ml	Structural	
2	CD9	145Nd	Novus	NB500-327	2.5 µg/ml		
3	CD98	173Yb	DVS-Fluidigm	3173018D	0.5 µg/ml		
4	Pan-Cytokeratin	148Nd	DVS-Fluidigm	3148022D	0.25 µg/ml	Epithelial	Phenotypic
5	E-cadherin	158Gd	DVS-Fluidigm	3158029D	0.25 µg/ml		
6	CD227/Mucin 1	150Nd	DVS-Fluidigm	3150032D	10 µg/ml		
7	EpCAM	144Nd	BETHYL	A700-077CF	25 µg/ml		
8	α-SMA	141Pr	DVS-Fluidigm	3141017D	10 µg/ml	Mesenchymal	
9	vimentin	143Nd	DVS-Fluidigm	3143029D	0.25 µg/ml		
10	N-cadherin	169Tm	BETHYL	A700-097CF	0.5 µg/ml		
11	integrin beta 1/CD29	160Gd	Novus	NBP2-22191	2.5 µg/ml		
12	P-p38	152Sm	Abcam	ab236527	0.5 µg/ml	Functional	
13	P-JNK	170Er	Invitrogen	# 700031	2.5 µg/ml		
14	P-ERK	171Yb	DVS-Fluidigm	3171021D	0.25 µg/ml		
15	β-catenin	165Ho	DVS-Fluidigm	3165032D	0.5 µg/ml		
16	SNAI1	161Dy	ThermoFisher	14-9859-82	0.25 µg/ml		
17	twist1/2	175Lu	Novus	HO117581-M01	0.25 µg/ml		
18	Zeb1	156Gd	Novus	NBP2-81015	0.25 µg/ml		
19	MMP9	162Dy	Novus	NBP2-80855	2.5 µg/ml		

Table S1. Antibody panel for FFPE imaging mass cytometry.
